# Skin aging: mechanisms, evaluation, and rejuvenation

**DOI:** 10.1038/s44318-026-00810-3

**Published:** 2026-05-21

**Authors:** Runhan Li, Jingyun Zhang, Kehang Mao, Dawei Meng, Jing-Dong J Han

**Affiliations:** 1 Peking-Tsinghua Center for Life Sciences, Academy for Advanced Interdisciplinary Studies, Center for Quantitative Biology (CQB), Peking University, Beijing, 100871 China; 2 Department of Biliary-Pancreatic Surgery, Renji Hospital Affiliated to Shanghai Jiao Tong University School of Medicine, Shanghai, 200127 China; 3 Peking University Chengdu Academy for Advanced Interdisciplinary Biotechnologies, Chengdu, China

**Keywords:** Skin, Stem Cells & Regenerative Medicine

## Abstract

Skin aging, the most visible and accessible manifestation of organismal aging, reflects systemic physiological decline, compromising barrier integrity, immune defense, and regenerative capacity—functions essential for overall tissue homeostasis and longevity. Understanding why and how the skin ages offers crucial insights into tissue homeostasis and systemic aging. Here, we dissect the multi-layered mechanisms of skin aging across the epidermis, dermis, and appendages, highlighting how intrinsic cellular senescence, disrupted inter-compartmental communication, and dysregulation of the skin microbiome and hormonal signaling collectively undermine epithelial structure and function. We also summarize advances in quantitative evaluation of skin aging, from molecular signatures to morphological, microbial, and phenotypic indices, enabling objective assessment of biological age and intervention efficacy. Finally, we highlight rejuvenation strategies, encompassing rewiring of gene expression programs, metabolic modulation, microenvironmental remodeling, microbiome modulation, and hormone regulation, offering a framework for precision interventions and next-generation regenerative therapies.

## Abstract (web summary)

This review examines epidermal and dermal aging as a systemic shift in tissue homeostasis, and suggests strategies for its assessment and multi-level stabilization to enhance skin resilience and healthspan.

## Graphical abstract

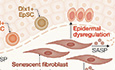

## Introduction

Skin aging is a multifactorial and dynamic process that influences not only local tissue homeostasis but also the physiology of the entire organism. As the body’s largest organ, the skin performs essential functions such as barrier protection, immune surveillance, sensory perception, and thermoregulation (Kubo et al, 2009; McArthur et al, 1998; Nolte et al, 1993; Shibasaki et al, 2006). With age, these functions deteriorate, compromising local tissue integrity and potentially propagating aging signals to distant organs (Bowden and McNulty, 2013; Coull et al, 2021; Hasegawa et al, 2020; Luebberding et al, 2013; Vu et al, 2022). Crucially, emerging experimental evidence positions the skin not merely as a victim of aging, but as an active participant in systemic aging. Experimental evidence shows that transplantation of senescent fibroblasts into the dermis of young mice triggered an increase in senescence markers in distal tissues, induced frailty, musculoskeletal decline, and cognitive impairment, thereby indicating that aged skin can propagate aging signals beyond the local tissue (Franco et al, 2025). Importantly, skin aging is embedded in a multi-organ network. Skin aging drives age-related bone loss via cystatin-A secretion (Liang et al, 2022a). In parallel, the skin interacts with the gut microbiota: fecal transplants from aged or young mice modulate both gene expression critical for skin function and physiological traits such as hydration and barrier integrity (Kim et al, 2022c; Tabata et al, 2024; Yu et al, 2024a). Understanding why and how the skin ages—not just locally but as a systemic regulator of functional decline—can guide interventions to preserve both appearance and overall healthspan (Baker et al, 2011; Ocampo et al, 2016).

This review first explores the multi-layered cellular and molecular mechanisms of aging across the epidermis, dermis, and appendages, highlighting how a breakdown in inter-compartmental communication undermines the skin ecosystem’s integrity. We then examine current approaches for assessing skin aging at multiple scales, from molecular signatures to tissue-level phenotypes, framing these aging clocks as integrated system readouts of the skin’s biological state. Finally, we highlight recent advances in rejuvenation strategies to reverse age-related changes, encompassing transcriptional, metabolic, microenvironmental, microbial, and hormonal interventions that aim to reprogram and restore youthful cellular function and systemic tissue resilience.

## Systemic deterioration of the skin ecosystem: why does the skin age?

**Figure 1:**
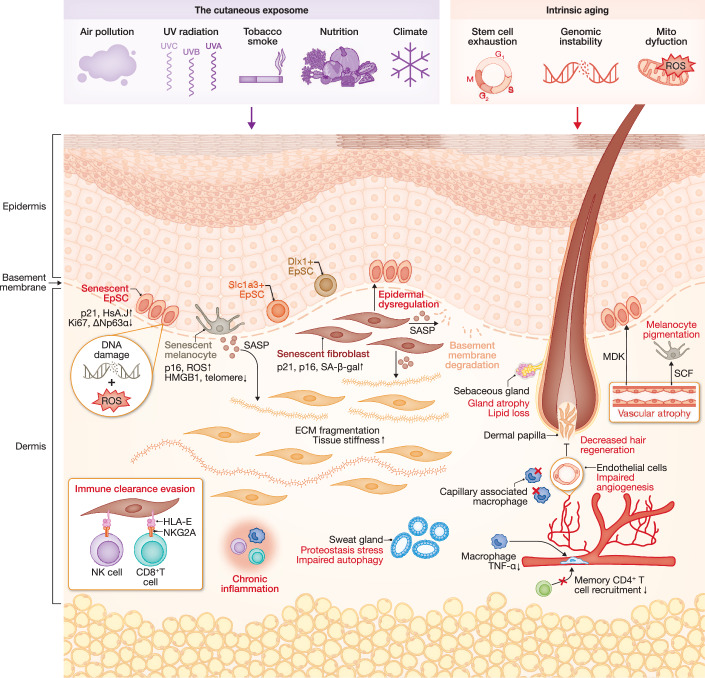
Systemic deterioration of the skin ecosystem during aging. Schematic cross-section of aged human skin illustrating how intrinsic aging mechanisms (stem cell exhaustion, genomic instability, and mitochondrial dysfunction) converge with the cutaneous exposome (air pollution, ultraviolet radiation, tobacco smoke, nutritional factors, and climatic stressors) to disrupt intercellular communication and tissue homeostasis (top). These combined inputs promote multicellular senescence and microenvironmental remodeling—characterized by SASP signaling, basement membrane degradation, ECM fragmentation/stiffening, chronic inflammation, vascular dysfunction, and impaired appendageal regeneration—culminating in tissue-level functional decline across the epidermis, dermis, and skin appendages.

**Figure 2:**
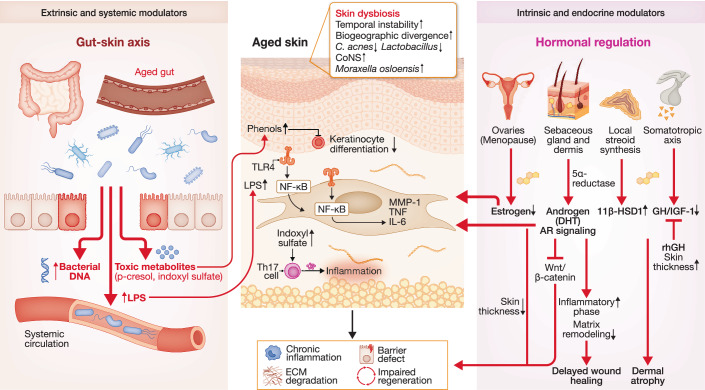
Converging gut–skin microbiome and endocrine mechanisms in skin aging. The figure is arranged as three framed panels to emphasize how extrinsic/systemic and intrinsic/endocrine modulators converge on shared tissue outcomes. Left (Extrinsic and systemic; gut–skin axis): age-associated gut dysbiosis and barrier leakiness promote systemic dissemination of microbial products (e.g., lipopolysaccharide (LPS), bacterial DNA) and metabolites (e.g., p-cresol, indoxyl sulfate), which are strongly shaped by exposome inputs (diet, medications, smoking/pollutants). Middle (Aged skin): circulating cues activate pattern-recognition pathways (e.g., TLR4) in keratinocytes and fibroblasts, driving NF-κB–linked inflammation and matrix metalloproteinase activity (e.g., MMP-1), impairing differentiation and accelerating extracellular matrix (ECM) breakdown; skin dysbiosis may further amplify these responses. Right (Intrinsic and endocrine; Hormonal regulation): reduced estrogen and GH/IGF-1 weaken collagen/skin thickness and regenerative capacity, while increased local androgen signaling (5α-reductase–dependent DHT/androgen receptor (AR)) antagonizes Wnt/β-catenin programs and delays wound healing; elevated 11β-HSD1 enhances glucocorticoid activation and dermal atrophy. Together, these axes bridge intrinsic aging and exposome inputs to drive chronic inflammation, barrier defects, and impaired repair.

Skin aging reflects the coordinated decline of a multi-compartmental ecosystem encompassing epidermis, dermis and appendages. Conceptually, this process is driven by the convergence of two distinct yet synergistic axes: intrinsic aging mechanisms and the cutaneous exposome (Naharro-Rodriguez et al, 2025). Intrinsic factors—such as stem cell exhaustion, genomic instability, epigenetic drift, and mitochondrial dysfunction—interact with a broad spectrum of extrinsic stressors. Importantly, the cutaneous exposome extends beyond well-characterized ultraviolet (UV) radiation to include particulate air pollution, tobacco smoke, and nutritional or climatic stressors (Fig. 1). Collectively, these forces disrupt intercellular communication and tissue homeostasis. A central consequence of this convergence is progressive remodeling of the cutaneous microenvironment. These local alterations are further shaped by systemic regulators, notably skin microbiome dysregulation and perturbations of the gut–skin axis, as well as age-associated hormonal changes (Fig. 2). This systemic imbalance manifests as impaired regeneration, extracellular matrix degradation, chronic inflammation, and functional deterioration of skin appendages (Arnal-Forné et al, 2024; Ezure et al, 2021; McCabe et al, 2020; Qin et al, 2017). Therefore, skin aging is not merely the sum of individual cellular changes but the outcome of interconnected failures spanning molecular, tissue, and systemic levels. Understanding this ecosystemic collapse provides the necessary framework for developing systems reprogramming strategies aimed at restoring youthful functional integrity.

### Epidermis and skin appendages aging

#### Keratinocyte and stem cell dysfunction

Keratinocytes, which constitute ~90% of the epidermis, maintain barrier integrity through continuous proliferation and differentiation driven by epidermal stem cells (EpSCs) in the basal layer (Tang et al, 2024a). Aging disrupts this homeostasis through intrinsic stem cell exhaustion and extrinsic environmental damage. In homeostasis, the interfollicular epidermis is sustained by molecularly and spatially distinct basal progenitor pools that can be genetically marked by *Dlx1*^*CreER*^ and *Slc1a3*^*CreER*^, respectively (Sada et al, 2016). Notably, the spatial organization of these basal domains in mouse skin shows parallels to human rete ridge and inter-ridge territories. This binary architecture imposes differential environmental exposure across progenitor niches; more deeply embedded progenitors (such as the *Slc1a3⁺* populations) are proposed to be relatively protected from UV-associated stress compared to their shallower counterparts (Ghuwalewala et al, 2024; Ghuwalewala et al, 2022; Wang et al, 2020). However, in chronologically aged interfollicular epidermis, basal cell proliferation declines, and EpSC heterogeneity diminishes—fast-cycling (*Slc1a3⁺*) clones are depleted, whereas slow-cycling (*Dlx1⁺*) populations prevail, collectively impairing regenerative capacity (Kwon et al, 2008; Raja et al, 2022). Concurrently, chronologically aged keratinocytes exhibit elevated p21 and the histone variant H2A.J, alongside reduced Ki67 expression and ΔNp63α downregulation, reflecting proliferative arrest (Kuang and Li, 2023; Rivetti di Val Cervo et al, 2012; Rübe et al, 2021) (Fig. 1).

Extrinsic stressors, particularly UV radiation, accelerates keratinocyte senescence through DNA damage and excessive generation of reactive oxygen species (ROS) (Lin et al, 2025a; Marrot and Meunier, 2008) (Fig. 1). In this context, senescent keratinocytes acquire a senescence-associated secretory phenotype (SASP), secreting matrix metalloproteinases (MMPs), IL-1α, IL-6, and TNF-α, which collectively degrade the extracellular matrix (ECM) and sustain chronic inflammation (Bashir et al, 2009; Chung et al, 1996; Dai et al, 2025; Dong et al, 2008). These intrinsic and extrinsic processes converge at the tissue level: aged human epidermis commonly exhibits epidermal thinning and progressive flattening of the dermal–epidermal junction (DEJ) with loss of rete ridges, reducing the exchange surface area and contributing to mechanical fragility and delayed repair (Branchet et al, 1990; Rittié and Fisher, 2015; Roig-Rosello and Rousselle, 2020). Functionally, even when baseline permeability measures (such as transepidermal water loss, TEWL) appear modestly changed, aged skin is more readily perturbed and shows delayed barrier recovery after disruption, consistent with compromised regenerative and lipid/lamellar body programs (Ghadially et al, 1995).

#### Melanocyte senescence and dysregulation

Epidermal melanocytes reside in the basal layer, where they produce melanin-containing melanosomes and transfer them to surrounding keratinocytes via dendritic processes, forming a supranuclear cap that absorbs UV radiation and prevents DNA mutations (Cichorek et al, 2013). Chronological aging drives melanocyte senescence, characterized by p16^INK4A^ upregulation, HMGB1 depletion, and telomere dysfunction (Victorelli et al, 2019). This senescence, together with a gradual decline in melanocyte density, underlies the uneven pigmentation observed in aged skin (Ortonne, 1990) (Fig. 1).

UV radiation further accelerates premature melanocyte senescence by forming pyrimidine dimers and other forms of DNA damage. This process is exacerbated by ROS generated during the photo-oxidation of melanin itself (Jenkins and Grossman, 2013). UV-induced senescence is also accompanied by metabolic change, including a shift toward glycolysis and mitochondrial dysfunction, which further increases ROS levels (Park et al, 2023b). Furthermore, the melanosome transport function of senescent melanocytes is impaired, leading to intracellular melanin accumulation and reduced transfer to keratinocytes (Park et al, 2023b).

Senescent melanocytes typically adopt a SASP, secreting factors such as IL-6, MMP-1, CCL2, and CXCL1 that degrade the ECM and inhibit keratinocyte proliferation, thereby contributing to epidermal thinning and chronic inflammation (Bandyopadhyay and Medrano, 2000; Park et al, 2023b; Victorelli et al, 2019) (Fig. 1). Interestingly, in nevi, dermal-clustered senescent melanocytes secrete osteopontin (SPP1), which activates dormant hair follicle stem cells (HFSCs) and robustly enhances hair regeneration and growth (Wang et al, 2023b).

#### Hair follicle, sebaceous gland, and sweat gland aging

Hair follicles are cyclic skin appendages that undergo lifelong transitions through growth (anagen), regression (catagen), and rest (telogen). HFSCs reside in the bulge niche and coordinate with mesenchymal and immune compartments to drive cyclical regeneration. Under injury or oncogenic stress, lineage boundaries relax, and HFSCs exhibit context-dependent plasticity, transiently contributing to the epidermis (Ge et al, 2017). During aging, HFSCs persist but enter deeper quiescence and exhibit delayed responses to activation cues. Sustained BMP signaling and elevated NFATc1 activity prolong telogen and postpone anagen entry (Keyes et al, 2013). Concurrently, accumulated DNA damage induces proteolysis of COL17A1, weakening HFSC anchoring, promoting aberrant upward differentiation, and ultimately depleting the stem cell pool (Matsumura et al, 2016). Beyond the bulge, early hair graying correlates with selective loss of matrix transit-amplifying progenitors and activation of the p53 pathway, which suppresses energy metabolism and cell proliferation (Wu et al, 2022). Telomere attrition represents a key source of this intrinsic genomic damage, directly linking telomere length to appendage dysfunction. Late-generation *mTR*^*−/−*^ mice with critically short telomeres develop profound hair/skin degenerative phenotypes (Rudolph et al, 1999), whereas epithelial *Tert* gain-of-function promotes progenitor activity and telogen-to-anagen transition (Flores et al, 2005; Sarin et al, 2005), and conditional telomerase reactivation can partially reverse degenerative changes (Jaskelioff et al, 2011; Siegl-Cachedenier et al, 2007). In humans, dyskeratosis congenita, a telomere biology disorder, provides a clinical correlate with mucocutaneous abnormalities (including reticulate pigmentation changes) and can include premature hair graying and hair loss (Savage, 2022). Compounding these intrinsic genomic deficits, extrinsic niche deterioration further exacerbates HFSC decline. Single-cell and live imaging studies demonstrate that reduced expression of adhesion and ECM genes—regulated by FOXC1 and NFATc1—facilitates HFSC migration out of the bulge (Zhang et al, 2021). This aged phenotype is reinforced by epigenetic drift: chromatin accessibility declines at promoters essential for self-renewal and differentiation, including bivalent sites, thereby locking HFSCs in a nonresponsive state (Koester et al, 2021). Consistently, maintenance of DNA methylation is critical, as epidermal *Dnmt1* deletion reduces HFSC activation probability and leads to progressive alopecia (Li et al, 2012).

Finally, emerging work suggests that age-associated epigenetic drift and inflammation may intersect with the loss of silencing at transposable elements (TEs), including endogenous retroviruses (ERVs), thereby activating antiviral pattern-recognition pathways and amplifying type I interferon–linked innate immune signaling via “viral mimicry” (Chiappinelli et al, 2015; De Cecco et al, 2019; Roulois et al, 2015). While direct evidence in human physiological skin aging remains limited, a recent mechanistic study demonstrated that the loss of a single histone methyltransferase (*Setdb1*) in adult murine skin aberrantly activates TEs. This epigenetic disruption alters hair follicle stem cell activation patterns and induces regenerative failure characterized by stem cell exhaustion and hair loss, aspects of which are remarkably reversible with antiviral intervention (Lyu et al, 2024). Consistent with the inflammation-centric features of aged skin, repetitive element and ERV dysregulation has also been directly implicated in chronic inflammatory skin diseases such as psoriasis (Krishnan & Kõks, 2023; Molès et al, 2005). This supports a plausible interface between chronic inflammation and TE control, providing a novel conceptual framework for understanding epidermal “inflammaging” and stem cell decline.

Sebaceous glands (SGs) are holocrine glands of the pilosebaceous unit, closely linked to hair follicle morphogenesis and maintained by dedicated local progenitor pools under Notch regulation (Andersen et al, 2019; Veniaminova et al, 2019). During aging, SGs follow a trajectory from initial hyperplasia in sun-exposed regions to eventual atrophy, accompanied by a decline in sebum production (Hou et al, 2022) (Fig. 1). These age-related changes are driven by both intrinsic cellular senescence and external stressors. Mechanistically, persistent p53 activation depletes *Blimp1*^*+*^ progenitors and impairs their renewal, accelerating SG loss (Kim et al, 2014). Wnt/β-catenin signaling limits terminal sebocyte differentiation. Age- or context-dependent shifts in Wnt activity, together with antagonism between the androgen receptor (AR) and β-catenin, bias sebocyte fate toward proliferation rather than holocrine maturation (Kretzschmar et al, 2015; Niemann, 2009). This shift contributes to both sebaceous hyperplasia and reduced lipid production. Extrinsic aging factors, including UV exposure and endocrine-disrupting pollutants such as dioxins and polycyclic aromatic hydrocarbons, activate aryl hydrocarbon receptor (AhR) signaling in human sebocytes. This suppresses lipogenesis and redirects sebaceous progenitors toward a keratinocyte-like fate, accompanied by IL-6-mediated chronic inflammation, ultimately depleting mature sebocytes and reducing sebum production (Hu et al, 2016a; Hu et al, 2016b; Ju et al, 2011).

Eccrine sweat glands (SWGs) are simple coiled tubular appendages essential for thermoregulation. With aging, both the number and total volume of SWG clusters decline, accompanied by morphological reorganization (Vilches et al, 2002; Zonnefeld et al, 2024). Dermal thinning contributes to these changes by pushing the secretory coil upward, resulting in increased ductal tortuosity and rotation (Ezure et al, 2021). These structural shifts coincide with functional decline, as older adults show lower regional sweat losses across most body sites under equivalent heat stress, indicating compromised thermoeffector capacity (Coull et al, 2021). Mechanistically, SWG aging is associated with proteostatic stress and impaired autophagy (Fig. 1). This is evidenced by the accumulation of enlarged p62/SQSTM1-positive protein aggregates, which are associated with reduced gland numbers in epithelial Atg7-deficient mice (Sukseree et al, 2018). In addition, aged SWGs exhibit an activated innate immune signature, characterized by the upregulation of antimicrobial and interferon-responsive genes (Tie et al, 2024). Their regenerative potential is also diminished: fewer glands in elderly skin produce cohesive keratinocyte outgrowths following injury, resulting in delayed re-epithelialization (Rittié et al, 2016).

### Dermis aging

#### Fibroblast senescence and ECM remodeling

The dermis, located directly beneath the epidermis, is critical for maintaining the skin’s structural and functional integrity. It is primarily composed of an ECM that is synthesized, organized, and remodeled by dermal fibroblasts (Plikus et al, 2021). With aging, fibroblast density and proliferative capacity decline, while p16^INK4a^-positive senescent fibroblasts accumulate, correlating with wrinkle severity and elastic fiber alterations (Fligiel et al, 2003; Varani et al, 2006; Waaijer et al, 2012) (Fig. 1). Concurrently, aged fibroblasts undergo a “cellular identity drift”, characterized by loss of functional priming, acquisition of adipogenic traits, and weakened intercellular crosstalk (Salzer et al, 2018; Solé-Boldo et al, 2020).

These phenotypic changes reflect underlying molecular mechanisms that arise from both intrinsic and extrinsic aging factors. Intrinsic drivers include persistent DNA-damage responses and mitochondrial oxidative stress, which activate p53-p21 and p16^INK4a^ programs, enforcing growth arrest (Zhang et al, 2024). These changes are reflected in classical senescence biomarkers: p53, p21, and SA-β-gal increase, whereas lamin B1 decreases in aged or UV-exposed fibroblasts (Chaiprasongsuk and Panich, 2022; Chen et al, 2008; Wang et al, 2017a). In parallel, mitochondrial ROS further engages AP-1 and JunB, suppresses IGF-1 signaling, and contributes to stem cell niche depletion and dermal atrophy (Maity et al, 2021). Extrinsic factors, particularly photoaging from UV radiation, amplify these mechanisms by reducing TGF-β receptor II (TβRII) and Smad signaling, leading to downregulation of CTGF and type I procollagen, thereby suppressing collagen synthesis (Quan et al, 2004).

Beyond classical DNA damage and ROS pathways, accumulating evidence indicates that metabolic reprogramming is a key upstream determinant of senescent fibroblast phenotypes and matrix remodeling. Metabolomic profiling of senescent human fibroblasts revealed increased glycolysis and pentose phosphate pathway activity, together with a characteristic extracellular “senescence metabolome” enriched for metabolites such as citrate and oxidative stress-related molecules, supporting a functional link between metabolic state and senescence-associated tissue phenotypes (James et al, 2015; James et al, 2018). Nutrient-sensing circuitry links this metabolic state to inflammatory secretomes: mTORC1 promotes IL-1α translation to sustain NF-κB-driven SASP programs, and rapamycin selectively blunts this pro-inflammatory output (Laberge et al, 2015). In parallel, NAD⁺ salvage metabolism (HMGA–NAMPT–NAD⁺ axis) governs the pro-inflammatory SASP independently of growth arrest via AMPK–p53–p38–NF-κB signaling, emphasizing that “senescence metabolism” actively shapes secretory and matrix-degrading programs (Nacarelli et al, 2019). Consistent with a bioenergetic “dial” on SASP composition, mitochondrial dysfunction–associated senescence (MiDAS) features a lower NAD⁺/NADH ratio and a modified SASP that lacks the IL-1–dependent inflammatory arm (Wiley et al, 2016).

Driven by these combined intrinsic, extrinsic, and metabolic alterations, accumulated senescent fibroblasts establish a potent pro-inflammatory SASP (Coppé et al, 2008). SASP factors, including IL-6 and MMPs (MMP-1 and MMP-9), dismantle the ECM by degrading collagen and fragmenting elastic fibers (Maity et al, 2021). This degradative activity is further exacerbated by an imbalance with endogenous inhibitors, as exemplified by the protective role of TIMP-1 in vivo (Qin et al, 2017; Yokose et al, 2012). Beyond direct matrix damage, senescent fibroblasts release extracellular vesicles containing SASP factors such as IL-6. These vesicles disrupt fibroblast-keratinocyte communication, impairing epidermal differentiation and barrier function, and amplify paracrine inflammation (Choi et al, 2020). Collectively, the convergence of reduced fibroblast number and altered identity, ROS-driven intrinsic senescence, metabolic dysregulation, UV-mediated suppression of TGF-β-dependent collagen anabolism, and SASP-directed ECM degradation leads to collagen fragmentation, dermal thinning, and impaired repair in aged skin (Fig. 1).

#### Endothelial dysfunction and vascular atrophy

The dermal vasculature is not merely a passive conduit but an active, zonated organ vital for skin homeostasis, thermoregulation, immune surveillance, and repair (Jonidi Shariatzadeh et al, 2025). It consists of endothelial cells (ECs) that regulate barrier integrity and vasomotion, and contractile pericytes that stabilize microvessels. Together, these components form a highly organized network of superficial and deep plexuses that generate capillary loops supplying the epidermis (Braverman, 2000).

With chronological aging, this vascular network undergoes marked structural and functional deterioration. A hallmark of this decline is capillary rarefaction, the progressive loss of microvessels and ECs, driven by diminished endothelial proliferative capacity (Chung et al, 2002; Wu et al, 2025). This process is further aggravated by a decline in the number and regenerative potential of pericytes (Zhuang et al, 2021). Functionally, thermoregulatory reflex vasoconstriction in response to cooling becomes blunted in aged skin, reflecting a reduced vasomotor reserve (Thompson and Kenney, 2004).

Extrinsic factors, particularly UV exposure, exacerbate vascular degeneration, leading to the formation of dilated and tortuous vessels embedded within solar elastosis and encased by multilaminated basement membranes. (Toyoda et al, 2001). Compounding these changes, the selective depletion of capillary-associated macrophages (CAMs) outpaces microvessel loss, creating macrophage-deficient niches that fail to clear luminal debris and further accelerate vascular rarefaction (Mesa et al, 2025) (Fig. 1).

#### Immunosenescence and chronic inflammation

The skin immune system, an indispensable component of host defense, comprises both resident cells, such as Langerhans cells (LCs), macrophages, mast cells, and tissue-resident memory T cells, and recruited circulating cells, including neutrophils and lymphocytes (Nguyen and Soulika, 2019). With advancing age, this system undergoes immunosenescence, a progressive functional decline that impairs wound healing, weakens antimicrobial defense, and increases susceptibility to skin diseases (Chambers and Vukmanovic-Stejic, 2020). Importantly, age-associated immune dysfunction in skin reflects not only cell-intrinsic changes but also a progressively altered cutaneous niche that can constrain immune cell positioning, activation and trafficking. Langerhans cells (LCs), the sentinel antigen-presenting cells of the epidermis, show a marked age-related decline in both number and functional capacity (Hasegawa et al, 2020). This decline is attributed to two interconnected mechanisms: first, impaired recruitment of monocyte precursors driven by reduced keratinocyte-derived CXCL14; and second, diminished migratory capacity resulting from decreased IL-1β levels (Cumberbatch et al, 2002; Hasegawa et al, 2020; Pilkington et al, 2018).

T cell–mediated immunosurveillance in aged skin is likewise increasingly constrained by a suppressive tissue milieu. Although varicella-zoster virus (VZV)-specific CD4⁺ T cells are enriched in human skin and remain functionally competent in older individuals, aged skin exhibits an increased local inhibitory tone, including higher Foxp3⁺ regulatory T cells and elevated PD-1 expression on cutaneous CD4⁺ T cells, which may restrain effective immune responses in situ (Vukmanovic-Stejic et al, 2015). While intrinsic factors such as reduced autophagic capacity (He et al, 2024b) may contribute to immune dysfunction, accumulating evidence indicates that age-related impairment of cutaneous recall responses is often rate-limited by the tissue microenvironment rather than a primary defect in memory T cell skin-homing or migratory potential. Specifically, defective activation of dermal blood vessels in older individuals—driven by reduced macrophage-derived TNF-α—restricts memory CD4⁺ T cell entry into antigen-challenged skin, and macrophage TNF-α production can be at least partially restored upon innate (TLR) stimulation, underscoring a reversible microenvironmental bottleneck (Agius et al, 2009) (Fig. 1). This microenvironmental suppression is further exacerbated by extrinsic stressors, which promotes expansion of regulatory T cells and perturbs local immune homeostasis (Yamazaki et al, 2014). Collectively, these changes bias aged skin toward a state of chronic, low-grade inflammation with impaired immune surveillance and delayed repair.

Consequently, there is growing interest in utilizing the tissue microenvironment to boost immune responses in aged skin. Reflecting this paradigm, recent therapeutic perspectives emphasize breaking the senescence–inflammageing–immune dysfunction cycle by targeting interactions between the altered tissue environment and immune cells (for example, senolytics and inflammation-modulating “senomorphics”), thereby restoring tissue immune surveillance and improving immune responsiveness during ageing (Bracken et al, 2025; Kirkland and Tchkonia, 2020; Wang and Nakanishi, 2025). Consistent with the concept that the aged niche can be therapeutically reprogrammed, early sterile inflammation and p38 MAPK–linked cytokine production in older skin are associated with attenuated VZV challenge responses, whereas short-term p38 inhibition (losmapimod) enhances antigen-specific cutaneous immunity in older individuals (Vukmanovic-Stejic et al, 2018).

### Disrupted crosstalk across epidermis, dermis, and appendages

Skin aging is fundamentally characterized by a progressive collapse of the multiscale ecosystem, driven by a breakdown in multicellular crosstalk that couples immune evasion, inflammatory signaling, aberrant mechanotransduction, and niche failure across dermal and epidermal compartments. The accumulation of senescent cells serves as a primary catalyst for this decline, fostering a pro-inflammatory and structurally compromised niche that propagates aging signals through the tissue (Furman et al, 2025).

Within this failing ecosystem, senescent fibroblasts in the dermis actively evade immune clearance by NK and CD8 + T cells through HLA-E upregulation (Pereira et al, 2019) (Fig. 1). These cells further destabilize the microenvironment by secreting CCL2 to recruit inflammatory monocytes, which release prostaglandin E₂ (PGE2) to suppress local T-cell memory and impair immune surveillance (Chambers et al, 2021). Simultaneously, in the epidermis, senescent melanocytes establish a self-reinforcing senescence loop via IP-10/CXCR3 signaling. This axis also induces “bystander” senescence in neighboring keratinocytes and fibroblasts through paracrine signals that elevate ROS and DNA damage, contributing to epidermal atrophy (Victorelli et al, 2019).

The integrity of the dermal niche is further eroded by the synergy of vascular decline and mechanical remodeling. UV-irradiated endothelial cells secrete stem cell factor (SCF), promoting melanocyte hyperpigmentation (Kim et al, 2018). Concurrently, age-related vascular atrophy increases dermal stiffness, which activates the mechanosensitive channel Piezo1 in epidermal stem cells, driving their premature differentiation (Ichijo et al, 2022). This stiffening also disrupts a critical fibroblast-endothelial-epidermal metabolic retinoid axis (MDK-SDC4-RBP1), impairing epidermal self-renewal (Wu et al, 2025) (Fig. 1).

The impact of ecosystemic deterioration is also evident in skin appendages, where the aged dermal niche dictates stem cell behavior (Ge et al, 2020). Mechanistically, increased matrix stiffness silences key HFSCs genes, inhibiting their activation (Koester et al, 2021). Within this failing niche, dermal papilla-vascular communication weakens, while macrophage-derived cytokines signal via JAK-STAT pathways to enforce quiescence and further suppress regenerative activity (Wang et al, 2019; Zhou et al, 2025b) (Fig. 1). Collectively, this intricate network of dysfunctional signaling drives the systemic breakdown of skin structure and function, reinforcing the need to view skin aging as a holistic system failure.

### Systemic regulators of skin aging

#### Skin microbiome dysregulation and gut–skin axis

The skin and gut host microbial communities that are essential for barrier integrity and immune function. These communities undergo age-dependent remodeling linked to biological frailty, a phenomenon particularly pronounced in the skin (Larson et al, 2022). Aging is marked by increased temporal instability and biogeographic divergence of the skin microbiota, characterized by depletion of *Cutibacterium acnes* and *Lactobacillus* alongside expansion of coagulase-negative staphylococci and other pathobionts (Howard et al, 2022; Larson et al, 2022; Swaney et al, 2025). These microbial shifts parallel host physiological aging, including reduced sebaceous gland area and altered skin-surface lipid composition (Howard et al, 2022; Swaney et al, 2025). Notably, specific taxa are predictive of distinct aging phenotypes: *Moraxella osloensis* is identified as a microbial marker of premature aging, experimentally shown to promote collagen catabolism and cellular senescence. Conversely, *Cutibacterium acnes* is associated with delayed aging, maintaining barrier integrity through lipid hydrolysis and acidification (Xia et al, 2024). Together, these findings highlight the skin microbiome as a modifiable target for interventions to mitigate age-associated dermatological decline.

Systemically, age-associated gut microbiome dysbiosis accelerates cutaneous aging by increasing circulating microbial products and noxious metabolites that reach the skin. Age-related gut dysbiosis coupled with increased intestinal permeability elevates systemic exposure to bacterial products such as lipopolysaccharide (LPS) (Fransen et al, 2017; Kim et al, 2016; Thevaranjan et al, 2017). In the skin, LPS engages TLR4 signaling on keratinocytes and dermal fibroblasts (Song et al, 2002; Yao et al, 2015), activating NF-κB-dependent expression of pro-inflammatory cytokines (TNF, IL-6) and MMP-1 (Cho et al, 2014; Kuzmich et al, 2017), thereby driving chronic inflammation and extracellular matrix degradation. Beyond endotoxin-driven inflammation, dysbiosis can shift microbial metabolism towards toxic by-products. Phenolic metabolites such as p-cresol can enter the circulation and impair keratinocyte differentiation, disrupting stratum corneum maturation and barrier function and predisposing to xerosis (Iizuka et al, 2009; Miyazaki et al, 2014). Gut-derived tryptophan catabolites can further amplify cutaneous inflammation, as indole-producing gut microbiota increases host indoxyl sulfate and activates AHR signaling in skin Th17 cells, with circulating indoxyl sulfate correlating with psoriasis severity in human cohorts (Wang et al, 2025a). In parallel, barrier dysfunction can permit translocation of microbes and their fragments into the bloodstream, and DNA of intestinal bacteria has been detected in patient plasma, supporting leakage of gut microbial material into systemic circulation (Ramírez-Boscá et al, 2015). Such microbial translocation and metabolite exposure may further disturb cutaneous immune homeostasis and perpetuate chronic inflammation, exacerbating age-related tissue damage. Causal evidence for this axis is provided by fecal microbiota transplantation (FMT): transferring microbiota from young to aged mice rejuvenates skin phenotypes, elevating circulating indoles and improving epidermal thickness and collagen abundance via aryl hydrocarbon receptor activation (Yu et al, 2024a). Together, these observations support a pro-aging gut–skin axis in which barrier dysfunction and dysbiosis-derived metabolites amplify cutaneous inflammaging, impair epidermal differentiation, and accelerate dermal matrix breakdown (Fig. 2).

#### Hormonal regulation of skin aging

Beyond gut-derived metabolites, the endocrine system represents a parallel regulatory axis. The skin is both a hormonal target and an active site of intracrine steroid metabolism. Thus, age-related hormonal shifts directly disrupt skin homeostasis through systemic decline and altered local hormone processing.

Estrogen acts as a pleiotropic regulator of cutaneous homeostasis by enhancing dermal vascularization and hydration, maintaining fibroblast viability and ECM integrity, promoting wound repair, and prolonging the anagen phase of hair follicle (Ashcroft et al, 1997; Barakat et al, 2016; Piérard-Franchimont et al, 1995; Zomer and Cooke, 2023). In women, the marked decline in circulating 17β-estradiol during the peri- and postmenopausal periods constitutes a major intrinsic driver of skin aging, leading to measurable reductions in collagen content and skin thickness (Brincat et al, 1987; Castelo-Branco et al, 1992; Sowers et al, 2008). Causality is further substantiated by animal models: ovariectomy accelerates intrinsic and UV-induced skin aging in mice (Tsukahara et al, 2004), while the specific deletion of estrogen receptor β (ERβ) disrupts collagen biosynthesis, confirming receptor-specific control over matrix turnover (Markiewicz et al, 2013).

Distinct from the protective role of estrogen, androgen signaling exerts complex, anatomic site-specific effects. At the tissue level, the skin is an important site of intracrine androgen activation: type 1 5α-reductase activity is enriched in sebaceous glands and exhibits marked regional differences, supporting heterogeneous local conversion of testosterone to dihydrotestosterone (DHT) across body sites and thereby shaping site-specific pilosebaceous programs (Thiboutot et al, 1995). Beyond adnexal structures, androgen receptor (AR) signaling contributes to dermal matrix homeostasis, as AR deficiency reduces skin collagen content (Markova et al, 2004). However, in the context of tissue repair, androgens become deleterious. AR activity is implicated in the age-associated delay in wound healing observed in males, mechanistically functioning by prolonging the inflammatory phase and dysregulating matrix remodeling (Ashcroft and Mills, 2002; Gilliver et al, 2007). Furthermore, at the stem cell level, AR acts as a negative regulator of regeneration by antagonizing the Wnt/β-catenin pathway, biasing lineage commitment and compromising appendage regeneration (Kretzschmar et al, 2015).

Beyond sex steroids, skin aging is accompanied by a systemic neuroendocrine shift, where anabolic support (growth hormone and insulin-like growth factor-1) wanes (Iranmanesh et al, 1991; Maggio et al, 2006), while catabolic stress signaling (glucocorticoids) and metabolic dysregulation (thyroid/insulin axes) increasingly compromise barrier integrity, ECM turnover, and regenerative capacity (Choi et al, 2006; Oikarinen et al, 1998; Okano et al, 2016). Notably, cutaneous glucocorticoid tone is governed by both systemic exposure and local regulation. Human scalp hair follicles exhibit an hypothalamic–pituitary–adrenal (HPA) axis–like system capable of cortisol production and feedback regulation (Ito et al, 2005), while human skin cells, including dermal fibroblasts, support local activation of glucocorticoids via 11β-HSD1–dependent intracutaneous cortisol generation (Tiganescu et al, 2011). With aging, increased 11β-HSD1 activity amplifies local glucocorticoid concentrations, driving dermal atrophy (Tiganescu et al, 2013). Additionally, the somatotropic axis declines with age, with clinical trials indicating that recombinant human growth hormone can restore skin thickness in elderly men, highlighting the multifaceted hormonal control of skin homeostasis (Rudman et al, 1990). Together, skin aging is driven by a coordinated shift in endocrine equilibrium (Fig. 2).

## Multiscale assessment of the skin ecosystem: how to quantify skin aging?

### Traditional methods

Traditional assessment of skin aging encompasses several interrelated aspects, including structural integrity, functional performance, and fluid biomarkers (Wang et al, 2025c) (Table 1). Structural assessment captures both the visual and morphological manifestations of aging, spanning superficial and deeper tissue changes. Superficial features include alterations in skin color and radiance, such as dullness and uneven pigmentation, as well as the formation of wrinkles, which serve as primary indicators for evaluating aging and the effects of interventions (Henseler, 2023; Meng et al, 2021; Pan et al, 2020; Panhard et al, 2012; Tsukahara et al, 2011; Turlier et al, 2021). Deeper structural changes involve loss of elasticity, thinning of the epidermis and dermis, collagen depletion, and hair thinning or graying (Branchet et al, 1990; Haytoglu et al, 2014; Marcos-Garcés et al, 2014; Panhard et al, 2012; Pena et al, 2022; Wang et al, 2023a). Beyond direct morphological changes, structural integrity is further compromised by the accumulation of advanced glycation end-products (AGEs) in the dermal matrix (Odetti et al, 1992). These stable products, formed via non-enzymatic glycation of long-lived proteins like collagen, lead to irreversible crosslinking that stiffens the skin and contributes to sallow discoloration (Corstjens et al, 2008; Fessel et al, 2014). Notably, the development of imaging technologies, such as skin autofluorescence (SAF), has enabled the non-invasive quantification of tissue AGEs in vivo (Birukov et al, 2021). As an accessible aging assessment technology, SAF serves as a biophysical proxy for cumulative metabolic and structural damage, bridging the gap between molecular glycation and visible phenotype (Koetsier et al, 2010; Majchrzak et al, 2022; Waqas et al, 2022). Together, these features underlie the visible signs of skin aging and can be quantitatively evaluated using imaging and morphometric techniques, enabling non-invasive characterization of structural decline.

**Table 1:** Overview of traditional methods for assessing skin aging.

Category	Measurements	Methods	Age-related changes	References
Structural assessments	Skin color, radiance, pigmentation	Skin color indices (hemoglobin, melanin and CIELAB color); Skin roughness	Dullness, uneven pigmentation, increased roughness	(Arnal-Forné et al, 2024; Henseler, 2023; Pan et al, 2020)
	Wrinkles (number, depth, volume)	Three-dimensional morphometric analysis; Echogenicity	Increased wrinkles, deeper furrows	(Akazaki et al, 2002; Henseler, 2023; Tsukahara et al, 2011)
	Epidermal and dermal thickness	High-frequency ultrasound; Optical coherence tomography; Histology	Thinning of epidermis and dermis	(Branchet et al, 1990; Haytoglu et al, 2014; Wang et al, 2023a)
	Collagen content	Histology; Biochemical assays; Reflectance confocal microscopy	Collagen depletion	(Marcos-Garcés et al, 2014; Pena et al, 2022)
	Hair density and pigmentation	Hair color, density, diameter, porosity and break stress	Hair thinning and graying	(Panhard et al, 2012; Turlier et al, 2021)
	Advanced glycation end-products (AGEs)	Skin autofluorescence	Accumulation of AGEs	(Koetsier et al, 2010; Majchrzak et al, 2022; Waqas et al, 2022)
Functional assessments	Skin barrier function	Transepidermal water loss (TEWL); Stratum corneum hydration; Surface pH	Decreased TEWL; Reduced stratum corneum; Slightly elevated pH	(Akdeniz et al, 2018; Man et al, 2009; Meng et al, 2021; Sato et al, 2014; Wilhelm et al, 1991)
	Sebum secretion	Sebum production	Decreased sebum secretion	(Lee et al, 2023; Meng et al, 2021)
	Elasticity	Cutometer; Mechanical testing	Reduced elasticity	(Ambroziak et al, 2019; Chen et al, 2024; Ryu et al, 2008; Vogel, 1987)
	Thermoregulation	Sweat secretion; Cutaneous blood-flow response	Reduced sweat secretion; Attenuated blood-flow reaction	(Ezure et al, 2021; Inoue & Shibasaki, 1996)
Fluid biomarkers	Skin wash fluids	EGF, FGF-2, IFNα2, IL-1RA, HSA, keratin-6, involucrin and cortisol	Cortisol increases with age; Others decline with age	(Kinn et al, 2015)
	SASP proteins	IL-8, IL-1β, IFN-γ, and MMPs secreted by dermal fibroblasts	Increase with age	(Waldera Lupa et al, 2015)
	Oxidative damage	8-OHdG	Positively correlated with skin-aging phenotypes	(Allerhand et al, 2011)
	Circulating marker	IGF-1	Negtively correlated with skin-aging phenotypes	(van Drielen et al, 2015)

This table summarizes major traditional approaches used to evaluate skin aging across structural, functional, and fluid biomarker dimensions.

*SASP* senescence-associated secretory phenotype, *MMP* matrix metalloproteinase.

Functional measurements primarily evaluate the skin’s ability to maintain barrier function and mechanical resilience. Barrier impairment, for instance, is reflected by reduced stratum corneum hydration, decreased transepidermal water loss, decreased sebum secretion, and slight elevation of surface pH (Akdeniz et al, 2018; Lee et al, 2023; Luebberding et al, 2013; Man et al, 2009; Paul et al, 2011). Declines in elasticity further indicate diminished tissue support and correlate strongly with wrinkle formation (Ambroziak et al, 2019; Ryu et al, 2008). Functional assessments also include thermoregulatory capacity, often demonstrated by reduced sweat secretion and attenuated cutaneous blood-flow responses in older adults (Ezure et al, 2021; Inoue and Shibasaki, 1996).

Fluid biomarker assessments provide additional mechanistic insight, linking structural and functional alterations to molecular aging. Key indicators detected in skin wash fluids—EGF, FGF-2, IFNα2, IL-1RA, HSA, keratin-6, and involucrin—decline with age, whereas cortisol levels increase (Kinn et al, 2015). In parallel, SASP proteins secreted by dermal fibroblasts (IL-8, IL-1β, IFN-γ, and MMPs) rise with age, highlighting increased inflammatory and senescence-associated activity within the dermis (Waldera Lupa et al, 2015). Furthermore, oxidative damage measured by 8-OHdG positively correlates with visible skin aging phenotypes, while circulating IGF-1 levels inversely associate with wrinkle severity, linking molecular alterations to skin structural decline (Allerhand et al, 2011; van Drielen et al, 2015).

Although traditional assessments capture the structural, functional, and biochemical manifestations of skin aging, they cannot fully represent the underlying biological heterogeneity of aging. Individuals of the same chronological age often display divergent skin aging trajectories due to genetic, lifestyle, and environmental influences. These limitations underscore the need for quantitative methods that move beyond descriptive evaluation and toward molecularly grounded, individualized measurement of aging.

Accurate quantification of skin aging is fundamental to understanding its biological mechanisms, monitoring interventions, and developing rejuvenation strategies. While chronological age provides a temporal reference, it fails to capture the heterogeneity of biological aging shaped by genetics, lifestyle, and environmental exposures (Han, 2024). Therefore, diverse molecular and phenotypic aging clocks have been developed to objectively measure the biological state of the skin across multiple scales—from epigenetic and transcriptomic landscapes to cellular morphology, microbiome composition, and visible appearance (Table 2). These quantitative frameworks not only provide biomarkers of aging but also serve as tools to evaluate therapeutic efficacy and to elucidate the interplay between intrinsic and extrinsic aging processes.

**Table 2:** Multimodal aging clocks related to skin aging: data types, sample sizes, models, and performances.

Data Type	Sample size	Model	Performance	Study
DNA methylation	7844 samples (51 tissues/cell types)	Elastic net regression (353 CpGs)	Training: *r* = 0.97, MAE = 2.9 years; Test: *r* = 0.96, MAE = 3.6 years	(Horvath, 2013)
DNA methylation	656 individuals (aged 19–101 years)	Elastic net regression (71 CpGs)	Cross validation: *r* = 0.91, MAE = 5.1 years	(Hannum et al, 2013)
DNA methylation	Training: 1119 samples; Test: 1112 samples	Elastic net regression (391 CpGs)	Pearson correlation *r* > 0.9 (blood/skin/saliva); *r* = 0.83 (neurons)	(Horvath et al, 2018)
DNA methylation	508 skin samples	Elastic net regression (2266 CpGs)	Training: RMSE = 2.34 years, MAE = 1.94 years; Testing:, RMSE = 3.89 years	(Boroni et al, 2020)
DNA methylation	378 female volunteers	Elastic net regression (1,000 CpGs)	Testing: MAE = 4–6 years	(Bienkowska et al, 2023)
DNA methylation	114 psoriatic, 41 uninvolved, 62 normal samples	Horvath’s multi-tissue clock (353 CpGs)	Spearman correlation *r* = 0.78–0.98	(Shen et al, 2018)
RNA-seq	133 healthy, 10 HGPS samples	Ensemble ML (ridge regression, random forests, gradient boosting)	MAE = 7.7 years, *R*² = 0.81	(Fleischer et al, 2018)
miRNA-seq	64 skin biopsies	Elastic Net regression, SVC classification	Regression: *R*² = 0.53, MAE = 10.89 years; Classification: Accuracy = 80.8%	(Roig-Genoves et al, 2024)
RNA-seq	887 samples	Pathway-based artificial neural network (Hallmark gene sets), ensemble learning	Test: MAE = 4.7 years	(Holzscheck et al, 2021)
DNA methylation	Pan-tissue: 6547; Fibroblast: 96; Plasma: 36	Hierarchical neural network on Reactome pathways	MAE = 2.83 years, Pearson correlation *r* = 0.97	(Prosz et al, 2024)
Biophysical assays	32 samples (aged 2–96 years)	Multivariate deterministic model (biophysical descriptors) with linear regression	MAE = 5.9 years	(Phillip et al, 2017)
16S rRNA-seq	1200 samples	Random forest, XGBoost, LightGBM	MAE = 6.36 years	(Carrieri et al, 2021)
Facial images	Image datasets (IMDB-WIKI)	VGG-16 CNN architecture pretrained on ImageNet, finetuned on IMDB-WIKI	MAE = 3.22 years	(Rothe et al, 2015)
Facial images	Image datasets (MORPH-II, FG-NET, MegaAge)	Attention-based Dynamic Patch Fusion (ADPF) framework with AttentionNet	MAE = 2.78 (MORPH-II); MAE = 4.65 (CACD); MAE = 4.71 (MegaAge-Asia)	(Wang et al, 2022)
Facial images	Image datasets (MORPH-II, MegaAge, CACD, CACDaAge)	FP-Age framework with pretrained face, parsing CNN	Superior MAE vs. SOTA	(Lin et al, 2025b)
Facial images	Image datasets (MORPH-II, FG-NET, Adience)	Improved Swin Transformer with attention-based convolution (ABC) module	MAE = 2.94 (MORPH-II); MAE = 5.28 (FG-NET); MAE = 3.85 (Adience)	(Shi et al, 2023)
3D facial images	~5000 Han Chinese	3D-based CNN	MAE = 2.8 years (chronological age); MAE = 4.1 years (perceived age)	(Xia et al, 2020)
Thermal facial images	>2800 thermal facial images	ThermoFace machine learning model (linear + CNN-based)	MAE = 5 years	(Yu et al, 2024b)
Dermoscopy images	Dermoscopy images from subjects of various ages	Linear regression on texture features	Demonstrating aging trends	(Choi et al, 2014)
Biophysical properties	300 healthy Korean women (aged 20–69 years)	Statistical regression analysis and five machine learning models	*R*^2^ = 0.79, RMSE = 6.22 yers	(Cho et al, 2022)
Facial expression videos	UvA-NEMO Smile Database (aged 8–76 years)	End-to-end CNN architecture	MAE = 4.74 years	(Pei et al, 2019)
Hand images	994 Indian women (aged 20–60 years)	Deep neural network	MAE = 5.89 years	(Georgievskaya et al, 2020)
Facial images	Image datasets (MORPH-II)	Mixture of deep networks (divide-and-conquer)	Superior MAE vs. SOTA	(Zhao et al, 2021a; Zhao et al, 2024)
Facial images	Image datasets (MORPH-II, FG-NET, MegaAge)	Knowledge distillation (ordinal/dark)	Superior MAE vs. SOTA	(Zhao et al, 2021a; Zhao et al, 2024)
Facial images	442,110 facial images	Deep learning-based age prediction	MAE = 6.61 years (IMDB-WIKI); MAE = 6.48 years (FG-NET);	(Király et al, 2025)

This table summarizes representative aging clocks across molecular, cellular, and phenotypic modalities, emphasizing approaches relevant to skin aging. Each model estimates biological age from molecular profiles, imaging data, or biophysical properties, with performance reported as mean absolute error (MAE), correlation coefficient (r), or coefficient of determination (*R*²).

*MAE* Mean Absolute Error, *MSE* Root Mean Squared 1109 Error, CpGCytosine–phosphate–Guanine dinucleotide, *ML* Machine Learning, *CNN* Convolutional Neural Network, *HGPS* Hutchinson–Gilford Progeria Syndrome, *SVC* Support Vector Classifier, *SOTA* State of the Art.

### DNA methylation clock

DNA methylation clocks, which track age-related changes in CpG methylation, have emerged as reliable tools for assessing skin aging. Horvath’s 2013 pan-tissue clock, based on 353 CpG sites, accurately predicts chronological age in epidermal samples (*r* = 0.96, error = 3.1 years), revealing age-dependent methylation shifts at Polycomb group target genes and other loci involved in epigenetic maintenance (Horvath, 2013). However, its accuracy in dermal fibroblasts is lower (error = 12 years), highlighting the need for skin- or cell-type-specific aging clocks. Hannum et al further advanced this field by profiling genome-wide methylation, identifying CpG sites associated with aging rates, laying the groundwork for skin-specific clocks (Hannum et al, 2013). Building on this approach, a dedicated skin-and-blood clock, using 391 CpG sites, improved accuracy (*r* = 0.91, error = 2.6 years) and detected accelerated aging in Hutchinson-Gilford Progeria Syndrome (HGPS) fibroblasts (Horvath et al, 2018). Subsequently, a skin-specific algorithm, trained on 2266 CpG sites, predicted age with high accuracy (*r* = 0.95, RMSE = 4.98 years) in external validation (Boroni et al, 2020). It highlighted enrichments in cAMP and calcium signaling pathways and detected a non-significant reduction in epigenetic age following rapamycin treatment of skin biopsies (68.0 vs. 70.4 years). More recently, the VisAgeX clock, which is based on large methylation regions, predicts visual skin aging with an MAE of 4–6 years. In addition to its predictive performance, pathway enrichment analysis further identified several biological processes linked to skin aging, including estrogen response, UV-responsive genes, hypoxia response, and epithelial–mesenchymal transition (Bienkowska et al, 2023). Notably, DNA methylation clocks remain stable under inflammatory conditions like psoriasis, where age-related CpG patterns are unaffected (*P* > 0.3), underscoring their robustness as aging biomarkers (Shen et al, 2018).

### Transcriptomic clock

Transcriptomic clocks, constructed from gene expression profiles, provide dynamic insights into the molecular processes underlying skin aging, complementing the relatively static nature of DNA methylation clocks. RNA-seq data from 133 human dermal fibroblast samples (aged 1–94 years, including HGPS donors) were used to develop an ensemble machine-learning model that predicted age with an overall median error of 7.7 years (*R*² = 0.81) and detected an apparent 9–10 year age acceleration in HGPS fibroblasts (Fleischer et al, 2018). Prediction errors in specific age ranges were reduced to approximately 4 years, demonstrating that fibroblast gene-expression profiles carry robust age-related signals, although they remain less precise than DNA methylation clocks in certain contexts. Likewise, a miRNA-based clock trained on 1856 miRNAs from healthy skin predicted biological age with a MAE of 10.89 years (*R*² = 0.53), performing robustly in epidermal samples even under disease contexts like psoriasis (Roig-Genoves et al, 2024). Although less precise than methylation or mRNA-based clocks, miRNA clocks capture an additional post-transcriptional regulatory layer, broadening the molecular dimensions of aging assessment.

Recent deep-learning frameworks have further refined transcriptomic aging prediction by incorporating biological pathway knowledge. A model leveraging 50 hallmark gene sets achieved a MAE of approximately 4.7 years across 887 epidermal samples, correlating with visible aging phenotypes and highlighting the p53 and TNFα/NFκB pathways as central drivers of photoaging (Holzscheck et al, 2021). Notably, these pathways are closely linked to canonical cellular senescence regulators, as p53 signaling is a key mediator of p21-dependent cell-cycle arrest, while NFκB signaling drives the SASP. This suggests that transcriptomic aging clocks may partially capture molecular programs associated with senescence, even though they do not directly measure classical senescence biomarkers such as p16, p21, or SA-β-Gal. Similarly, an explainable artificial intelligence model (XAI-AGE) integrating reactome pathway information detected partial age reversal in reprogrammed fibroblasts and complete “zero-age” resetting in induced pluripotent stem cells (iPSCs), implicating chromatin organization and circadian rhythm pathways as key regulatory axes (Prosz et al, 2024).

### Cellular morphology clock

Beyond molecular clocks, aging can be observed directly in cellular morphology and mechanics, capturing cellular-level changes that complement molecular markers. Age-related changes in the morphology and function of skin cells, especially fibroblasts and endothelial cells, are associated with reduced tissue regeneration and can serve as quantifiable markers of aging. Phillip et al conducted a comprehensive study on dermal fibroblasts from donors aged 2–96 years, combining morphological analysis (via high-throughput cell phenotyping, HTCP), biophysical/mechanical analysis (via microrheology and traction force microscopy), and biomolecular markers. Their findings revealed that aged cells exhibit increased cell size, nuclear irregularities, and stiffness, alongside reduced traction force and migration speed. Crucially, the morphology-based features alone (measured via HTCP) robustly predicted cellular age with high accuracy (best prediction pair: mean unbiased prediction error of 5.9 years) (Phillip et al, 2017). Advances in machine learning have enabled high-throughput and precise detection of these age-related features. For instance, a Cascade R-CNN model quantifies replicative senescence in fibroblast-like mesenchymal stem cells with 92% accuracy, identifying features like enlarged cell area, irregular nuclear shapes, and lysosomal expansion (He et al, 2024a). These morphological shifts, driven by cytoskeletal remodeling and organelle accumulation, link intracellular aging to functional decline in skin. By bridging molecular signatures, such as DNA methylation changes, with broader microenvironmental and phenotypic alterations, cellular morphology provides a functional readout of aging that can be quantified with imaging and machine learning, enabling high-throughput drug screening.

### Microbiome clock

The skin microbiome provides a complementary layer for aging assessment, reflecting interactions between host cells, environment, and microbial communities. AI analysis of 16S rRNA data from over 1200 leg skin samples reveals microbial shifts tied to aging phenotypes. Specifically, reductions in Cutibacterium and increases in Corynebacterium, Bacteroides, or Veillonella correlate with dryness, elasticity loss, and chronological age, allowing predictive models to estimate age with a MAE of approximately 6 years (Carrieri et al, 2021). Enrichment of Lactobacillus is indicative of younger, well-hydrated skin, whereas increased Bacillus abundance signals older, postmenopausal states. The reduction in beneficial taxa and enrichment of pathogenic or pro-aging genera can exacerbate extracellular matrix degradation and inflammation, accelerating skin aging. Building on this, AI-based analyses highlight phenotype-specific genera that may serve as targets for precision interventions.

### Phenotype clock

At the macroscale—visible to both the naked eye and the camera—AI enables non-invasive quantification of skin aging phenotypes such as wrinkles, pigmentary changes, volume loss, and elasticity decline. Deep learning methods applied to high-resolution facial images have achieved apparent-age prediction with low MAE. For example, the DEX model reaches an MAE of approximately 4 years on curated datasets (Rothe et al, 2015). Recent architectures employing attention-based dynamic patch fusion, focusing on periocular and nasolabial regions and face-parsing attention mechanisms, further improve patch-level precision, while enhanced Swin Transformer frameworks continue to refine performance (Lin et al, 2025b; Shi et al, 2023; Wang et al, 2022). Notably, the FaceAge model estimates biological age from facial images and demonstrates independent prognostic value across multiple cancer types, with patients appearing on average 4.79 years older than their chronological age. Importantly, FaceAge showed a significant association with the aging-related gene CDK6, suggesting that image-based aging predictors may partially reflect molecular pathways involved in biological aging and cellular senescence (Bontempi et al, 2025).

Multi-view 3D facial scans, enhanced by deep learning, quantify age-related skeletal and soft-tissue changes, such as widening of the mandibular angle and skin sagging in postmenopausal women, linking these alterations to life-history events like menopause and lifestyle factors (like smoking, sleep duration) (Xia et al, 2020). This 3D scan approach predicts biological age with high accuracy (MAE ~2.8 years), integrating genetic and environmental influences into a non-invasive phenotyping tool. Thermal imaging further enriches this framework by exploring physiological dimensions. For instance, the ThermoFace model predicts thermal age with an MAE of approximately 5 years, correlating with metabolic parameters, sleep duration, and DNA repair pathways (Yu et al, 2024b). Dermoscopy-based texture analysis further enables microstructural evaluation through wrinkle cell area and perimeter metrics validated across facial, neck, and hand regions (Choi et al, 2014).

Integrative models such as the skin age index (SAI) combine elasticity, wrinkles, and hydration measurements using a conditional random forest algorithm, achieving high determination coefficients and sensitivity to anti-aging interventions (Cho et al, 2022). Recent innovations broaden temporal and multimodal coverage: video-based apparent-age estimation captures dynamic expression cues, while hand-image analysis provides complementary insights into systemic aging (Georgievskaya et al, 2020; Pei et al, 2019). Mixture models and ordinal-relation learning enhance robustness across heterogeneous cohorts (Zhao et al, 2021a; Zhao et al, 2024). Large-scale cohort analyses reveal pronounced ethnic- and sex-specific aging trajectories, particularly postmenopausal facial changes (Flament et al, 2022). Moreover, recent work links facial age acceleration to mortality risk across occupations (Király et al, 2025).

Accurate skin aging clocks capture the biological state of the skin ecosystem across multiple scales, ranging from molecular signatures such as DNA methylation clocks, to cellular phenotypic clocks based on morphology and functional states, and ultimately to organism-level indicators including 3D facial phenotypes. Together, these complementary metrics create a comprehensive digital representation of the skin ecosystem, linking intracellular molecular hallmarks with macroscopic tissue and organismal features. Collectively, these approaches establish a largely non-invasive, multiscale phenotyping framework that enables longitudinal and interventional assessment of human skin aging. Importantly, such measurements allow dynamic monitoring of how the system deviates from youthful homeostasis, thereby providing quantitative baselines to identify precise intervention points for rejuvenation and cellular reprogramming strategies. Future integration of these diverse data streams through AI and machine-learning frameworks may further enable personalized skin-aging profiles and adaptive monitoring of rejuvenation interventions.

## Restoring the skin ecosystem: strategies for multi-level rejuvenation

**Figure 3:**
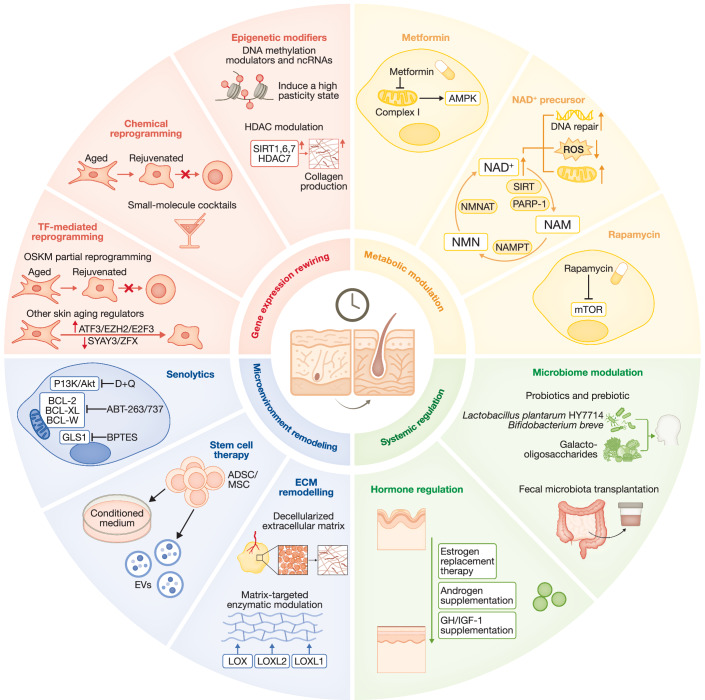
Rejuvenation strategies for skin aging. Schematic overview of major rejuvenation approaches targeting the hallmarks of skin aging. Gene expression rewiring through transcription factor (TF)-mediated reprogramming, chemical reprogramming, or epigenetic modifiers restores youthful gene expression programs. Metabolic modulation using metformin, rapamycin, or NAD⁺ precursors revitalizes mitochondrial function and energy homeostasis. Microenvironmental remodeling, including senolytics, stem cell-based therapy, and extracellular matrix (ECM) remodeling, rejuvenates the skin structure and function. Systemic regulation, encompassing microbiome modulation and hormonal regulation, further restores skin homeostasis by targeting organismal-level factors that influence cellular and tissue aging.

Skin aging reflects a coordinated decline of a multiscale ecosystem, driven by transcriptomic dysregulation, epigenetic drift, metabolic decline, and microenvironmental imbalance, as well as systemic microbial and hormonal dysregulation (An et al, 2025; Jankowski et al, 2025; Lee et al, 2021b; Martic et al, 2023; Zou et al, 2021). Rather than merely delaying deterioration, contemporary rejuvenation strategies aim to reverse these age-related alterations through systems reprogramming. This paradigm shift focuses on restoring youthful homeostatic equilibrium across molecular, cellular, and microenvironmental levels, while simultaneously recalibrating systemic axes to ensure tissue-wide resilience (Fig. 3). By framing rejuvenation as a holistic “reset” of the skin system, we can integrate diverse interventions—from cellular rewiring to microenvironmental remodeling—into a unified therapeutic roadmap.

### Rewiring of gene expression programs

Gene expression programs—defined by cell-type specific and spatiotemporally coordinated gene activation—constitute the blueprint for skin structure and function. Maintaining this transcriptional architecture is essential for epidermal renewal, extracellular matrix production, and barrier homeostasis (Köhler and Rodríguez-Paredes, 2020; Solé-Boldo et al, 2020). Aging progressively disrupts this blueprint through epigenetic drift and transcriptional dysregulation, leading to loss of transcriptional fidelity and increased noise (Enge et al, 2017; Martinez-Jimenez et al, 2017). Rewiring of gene expression programs can reverse age-associated alterations in transcriptional networks, chromatin accessibility, DNA methylation, and histone modifications, collectively restoring a youthful epigenetic and transcriptional landscape (Lu et al, 2020; Ocampo et al, 2016; Olova et al, 2019).

#### TF-mediated reprogramming

The discovery that a defined set of transcription factors—Oct4, Sox2, Klf4, and c-Myc (OSKM)—can reprogram somatic cells, including skin fibroblasts, into induced pluripotent stem cells (iPSCs) and even induced blastoids, revealed the remarkable plasticity of cell identity (Kurita et al, 2018; Liu et al, 2020; Liu et al, 2021b; Park et al, 2008; Takahashi et al, 2007; Yu et al, 2007). When applied transiently, OSKM induction does not erase lineage identity or increase the risk of teratoma formation but instead reverses multiple hallmarks of aging, a process known as partial reprogramming (Ocampo et al, 2016).

In human skin fibroblasts, partial OSKM reprogramming reduces both DNA methylation age and transcriptomic age, restores mitochondrial function, suppresses SASP gene expression (Sarkar et al, 2020). A refined approach, termed maturation phase transient reprogramming, can rejuvenate skin fibroblasts by approximately 30 years based on DNA methylation and transcriptomic clocks (Gill et al, 2022). Similarly, partial OSK reprogramming has been shown to reverse the DNA methylation age of human keratinocytes (Macip et al, 2024). In vivo, short-term cyclic OSKM induction in a progeria mouse model ameliorates age-associated skin phenotypes and extends lifespan (Ocampo et al, 2016). This rejuvenative effect was later validated in physiologically aged wild-type mice, where long-term partial OSKM reprogramming reversed the epigenetic age of skin, rejuvenated dermal architecture, and enhanced wound healing capacity (Browder et al, 2022). Mechanistically, these effects are associated with the reactivation of developmental regulators, suppression of inflammatory and stress-response pathways, restoration of youthful chromatin accessibility, and reduction of mesenchymal drift, collectively rewiring the gene expression network toward a more youthful configuration (Kim et al, 2025a; Lu et al, 2025; Sahu et al, 2024).

Beyond these pluripotency factors, targeting skin aging regulators can also rejuvenate the skin. For example, replenishing the age-related decline of ATF3 enhances basal stem cell proliferation and stimulates fibroblast collagen production by approximately 200% (Li, 2025; Luo et al, 2024). Moreover, systematic Perturb-seq screening of transcription factors identified that overexpression of EZH2 or E2F3, and repression of STAT3 or ZFX, confer rejuvenative effects on human skin fibroblasts, manifested as increased proliferation, diminished senescence, and improved proteostasis and mitochondrial activity (Sengstack et al, 2026). These findings highlight a promising route for selective, lineage-preserving transcriptional rejuvenation.

#### Chemical reprogramming

An alternative to genetic OSKM induction is chemical reprogramming, which employs defined cocktails of small molecules to modulate signaling pathways and epigenetic enzymes, thereby restoring youthful transcriptional and epigenetic states without genetic manipulation (Guan et al, 2022; Liuyang et al, 2023; Wang et al, 2025b). In skin fibroblasts, partial chemical reprogramming using a six- or seven-compound cocktail reverses multiple hallmarks of aging, including restoration of the transcriptomic and epigenetic clocks, reduction of aging-associated metabolites, and improvement of mitochondrial function (Mitchell et al, 2024; Yang et al, 2023). Another simplified two-compound cocktail has also been shown to effectively ameliorate age-related phenotypes in human skin keratinocytes and fibroblasts (Schoenfeldt et al, 2025).

Compared with genetic approaches, chemical reprogramming offers several advantages: it is highly controllable, non-integrative, and relies on small molecules that are easily synthesized, standardized, and tunable. This makes chemical reprogramming an attractive and potentially translatable strategy for skin rejuvenation through the reversal of aging-associated transcriptional and epigenetic alterations.

#### Epigenetic modifiers

Epigenetic drift, characterized by DNA methylation changes, histone modification imbalance, and 3D chromatin reorganization, is a hallmark of skin aging (López-Otín et al, 2023). Aging induce hypermethylation patterns in human skin, often in a locus-specific manner affecting promoters and enhancers linked to transcriptional regulation (Bormann et al, 2016; Grönniger et al, 2010; Kokikian et al, 2024; Raddatz et al, 2013). Reestablishing youthful chromatin dynamics represents a form of epigenetic reprogramming.

DNA methylation modulators such as 5-azacytidine can transiently erase epigenetic marks, inducing enhanced plasticity and rejuvenation-like states in skin fibroblasts (Manzoni et al, 2016; Mirakhori et al, 2015; Pennarossa et al, 2023a; Pennarossa et al, 2019). Notably, high-throughput screening has identified dihydromyricetin as a natural DNA methylation inhibitor that reduces epigenetic age and mitigates age-related epidermal thinning in human skin models (Falckenhayn et al, 2023). At the histone modification level, skin aging is characterized by a rise in histone acetylation coupled with a decline in the expression of specific histone deacetylases (HDACs), as observed in both UV-exposed and intrinsically aged human skin in vivo (Lee et al, 2021a). Pharmacological modulation of HDACs can attenuate these age-associated changes. For instance, phenol-croton oil peel has been shown to enhance type I and III collagen production by upregulating SIRT6 and SIRT7, members of the sirtuin family of NAD^+^-dependent histone deacetylases, thereby promoting a more youthful extracellular matrix (Cardoso et al, 2022). Similarly, shikimic acid protects against UV-induced skin senescence by activating SIRT1 (Martínez-Gutiérrez et al, 2021). Conversely, the downregulation of HDAC2 and HDAC7 has been demonstrated to induce senescence in dermal fibroblasts, while the re-expression of HDAC7 can delay cell-cycle arrest in pre-senescent cells, highlighting the critical role of specific HDACs in maintaining skin cell vitality (Warnon et al, 2021). Finally, noncoding RNAs also contribute to chromatin remodeling and cell identity maintenance. The miR-200 family, for example, synergizes with young extracellular matrix scaffolds to reverse skin fibroblast senescence and rejuvenate phenotypes (Pennarossa et al, 2023b). Thus, targeted manipulation of ncRNAs represents a next-generation strategy to restore youthful skin states. Collectively, these findings underscore that coordinated epigenetic interventions—including DNA methylation, histone modification, and ncRNA regulation—offer a promising avenue to modulate chromatin states and promote skin rejuvenation.

### Metabolic modulation

Aged skin exhibits profound metabolic alterations: reduced mitochondrial function, impaired autophagy, NAD⁺ depletion, and dysregulated nutrient-sensing pathways such as AMPK, mTOR (Ido et al, 2015; Kuehne et al, 2017; Miyamoto and Kudoh, 2013; Murase et al, 2020; Vidali et al, 2023). Metabolic modulation seeks to restore youthful energetic and signaling states through modulation of metabolic pathways. Three classic metabolic modulators—metformin, rapamycin, and NAD⁺ precursors—have emerged as leading candidates for skin rejuvenation through rebalancing metabolic states.

#### Metformin

Metformin, a classical AMPK activator, has shown potent effects in skin aging. In the epidermis, metformin protects against UVB-induced damage by suppressing ROS accumulation and apoptosis while dampening pro-inflammatory cytokine release, thereby alleviating photodamage in both human keratinocytes and UVB-exposed mouse skin (Ribeiro et al, 2020; Xiao et al, 2021). In dermal fibroblasts, it reverses high-glucose-induced senescence‑associated dysfunction via NF-κB inhibition, restoring extracellular matrix homeostasis (Soydas et al, 2021). Beyond cellular models, local metformin application accelerates wound healing in aged mice by enhancing epidermal regeneration, angiogenesis, and collagen deposition (Zhao et al, 2017). Furthermore, engineered extracellular vesicles loaded with metformin (Met-EVs) rejuvenate aged mouse skin repair by improving mitophagy and oxidative metabolism (Zhuang et al, 2024). In hair follicles, metformin activates AMPK signaling to induce anagen entry and enhance hair follicle regeneration ability, with clinical evidence of hair regrowth after topical use (Araoye et al, 2020; Chai et al, 2019; Sun et al, 2022). Strikingly, systemic metformin therapy decelerates the aging clock in male monkeys, reducing skin epigenetic age by 2.65 years and rejuvenating skin transcriptomes (Yang et al, 2024). Collectively, metformin integrates AMPK activation, mitochondrial restoration, and autophagy to re-establish youthful homeostasis and regenerative capacity in aged skin.

#### Rapamycin

Rapamycin, a canonical mTORC1 inhibitor, counteracts skin aging by restoring metabolic balance and cellular homeostasis. mTOR activation in aging drives anabolic overload, mitochondrial stress, and accumulation of senescent cells, leading to the SASP and ECM degradation (Chen et al, 2022; Cunningham et al, 2007; Laberge et al, 2015; Nacarelli et al, 2016). By restraining mTORC1 activity, rapamycin rebalances anabolic-catabolic fluxes, enabling cellular repair and resilience (Wang et al, 2017b).

At the cellular level, rapamycin attenuates oxidative and photoaging stress induced by H₂O₂, UVA, and UVB in dermal fibroblasts, which is achieved by reducing ROS, preserving type I collagen, downregulating p53, p21, and upregulating autophagy pathway (Bai et al, 2021; Li et al, 2024; Qin et al, 2018; Tang et al, 2024b). These mechanisms are supported by in vivo and clinical evidence: in mouse models, rapamycin mitigates UVB-induced damage via Hspb2-mediated autophagy and TGF‑β/Smad-dependent collagen preservation, and in a randomized human trial, topical rapamycin decreased p16^INK4A^ protein level, increased type VII collagen, and improved both histological and clinical features of aged skin (Chung et al, 2019; Li et al, 2024). Furthermore, rapamycin suppresses the SASP by reducing pro-inflammatory cytokines and the expression of p16 and p21 (Chung et al, 2019). Targeted rapamycin delivery strategies, such as CD9 monoclonal antibody-conjugated lactose-wrapped CaCO₃ nanoparticles, enhance cellular uptake and amplify anti-senescence effects (Thapa et al, 2017). Importantly, rapamycin also promotes regenerative processes: autophagy activation induces hair follicles to enter the anagen phase and stimulates hair growth (Wang et al, 2017b). In summary, by coordinating mTORC1 inhibition, autophagy activation, and SASP suppression, rapamycin restores proteostasis and reinstates regenerative potential in aged skin.

#### NAD^+^ precursor

Age-associated decline in nicotinamide adenine dinucleotide (NAD⁺) disrupts redox metabolism, mitochondrial function, and DNA repair, accelerating skin aging (Massudi et al, 2012; Miyamoto and Kudoh, 2013). Restoring the NAD⁺ pool through β-nicotinamide mononucleotide (NMN) or niacinamide (NAM) rejuvenates epidermal and dermal compartments by re-establishing energy homeostasis and stress resilience.

NMN acts as a direct biosynthetic intermediate in the NAD⁺ salvage pathway (Ryu et al, 2018). In murine UVB-induced skin damage, NMN supplementation preserves collagen and epidermal/dermal structure, reduces oxidative stress, and limits inflammation via NF-κB suppression (Zhou et al, 2021a; Zhou et al, 2021b). Mechanistic studies reveal that NMN activates NAD⁺/SIRT pathways, including SIRT3-mediated mitophagy, enhances mitochondrial proline biosynthesis for collagen production, and recruits glutathione to strengthen GPX4-mediated ferroptosis defense (Feng et al, 2022; Sun et al, 2025; Xu et al, 2025; Zhang et al, 2025). Beyond structural preservation, NMN promotes hair follicle health by reversing dihydrotestosterone-induced follicular atrophy, reducing oxidative stress, and inhibiting NF-κB in human dermal papillary fibroblasts (Xu et al, 2024). In aged melanocytes, NMN downregulates cAMP/Wnt signaling to decrease hyperpigmentation (Brito et al, 2022). Additionally, NMN protects dermal fibroblasts from particulate matter-induced senescence by enhancing Nrf2 and SIRT1 activity while reducing NF-κB-driven inflammation, demonstrating broad cytoprotective and regenerative effects across skin cell types (Chang et al, 2022).

NAM, also a widely used NAD⁺ precursor, exerts protective effects against extrinsic skin aging induced by environmental stress. NAM reduces ROS, enhances DNA repair, and diminishes SASP-related inflammatory signaling in human keratinocytes, fibroblasts, and melanocytes exposed to UV radiation or oxidative stress (Bierman et al, 2020; Camillo et al, 2022a; Camillo et al, 2022b; Chhabra et al, 2019; Surjana et al, 2013; Tan et al, 2022). NAM also contributes to the maintenance of keratinocyte homeostasis by preserving the balance between proliferation and differentiation (Tan et al, 2019). Clinical trials confirm that topical NAM reduces wrinkles, pigmentation, erythema, and transepidermal water loss, while improving elasticity and hydration, demonstrating efficacy in aged human skin (Bissett et al, 2004; Bogdanowicz et al, 2024; Vergilio & Leonardi, 2025). By replenishing NAD⁺ pools, NMN and NAM reactivate mitochondrial metabolism, enhance DNA repair capacity, and attenuate oxidative and inflammatory aging signatures.

### Microenvironment remodeling

The skin microenvironment is a dynamic ecosystem comprising fibroblasts, keratinocytes, immune cells, endothelial cells, and ECM, which collectively orchestrate tissue renewal, barrier function, and stress responses (Hur, 2024; Jevtić et al, 2020; Park et al, 2025; Quan et al, 2013). With aging, this network deteriorates through senescent cell accumulation, chronic inflammation, and structural disorganization (Ge et al, 2020; McCabe et al, 2020). Microenvironment remodeling aims to restore a youthful equilibrium by targeting cell composition, intercellular signaling, and matrix organization.

#### Senolytics

Accumulation of senescent cells in the skin drives chronic inflammation, ECM degradation, and impaired regenerative capacity (Malaquin et al, 2013; Samdavid Thanapaul et al, 2022; Victorelli et al, 2019). By selectively eliminating senescent cells, senolytics have emerged as a promising strategy to rejuvenate skin by restoring skin cell function and tissue homeostasis.

Dasatinib and quercetin (D + Q) act synergistically to induce apoptosis in senescent human dermal fibroblasts, suppress SASP factors, and increase collagen density in aged skin grafts (Takaya and Kishi, 2024). In vitro depletion of senescent dermal papillary fibroblasts using D + Q reverses SASP-mediated inhibitory interactions, restoring hair follicle inductive capacity (Pappalardo et al, 2025). These studies highlight the dual potential of D + Q to rejuvenate both dermal structure and skin appendage function. Moreover, BCL-2 family inhibitors such as ABT-263 and ABT-737 selectively induce apoptosis in senescent cells by targeting the anti-apoptotic proteins BCL-W and BCL-XL (Yosef et al, 2016). In aged murine and human-mouse chimeric skin, ABT-263 and ABT-737 selectively eliminate senescent dermal fibroblasts, leading to reduced expression of SASP factors (e.g., MMPs, IL-6), along with increased collagen density, epidermal thickness, and keratinocyte proliferation (Kim et al, 2022a; Kim et al, 2022b; Takaya et al, 2023). Topical administration of ABT-263 in aged mice further accelerates wound healing (Shvedova et al, 2024). Moreover, ABT-263 and ABT-737 trigger caspase-dependent apoptosis in UV-induced senescent melanocytes (Kim et al, 2025b), underscoring their potential to rejuvenate skin by clearing senescent cells across multiple compartments. Finally, glutaminase inhibitors, such as BPTES (bis-2-(5-phenylacetamido-1, 3, 4-thiadiazol-2-yl)ethyl sulfide), selectively clear senescent dermal fibroblasts in human-mouse chimeric models, leading to increased collagen density, enhanced dermal proliferation, and sustained suppression of SASP factors (Takaya et al, 2022). Collectively, these senolytics demonstrate that targeted clearance of senescent cells restores ECM integrity, suppresses inflammatory SASP signals, and improves both structures and functions of aged skin. Future directions include optimizing delivery methods, minimizing off-target toxicity, and combining senolytics with ECM remodeling or stem cell therapies to achieve comprehensive skin rejuvenation.

#### Stem cell therapy

Stem cell-based interventions rejuvenate aged skin primarily through paracrine signaling and extracellular vesicles (EVs), leading to microenvironmental remodeling. Adipose-derived stem cell (ADSC) interventions rejuvenate skin through multiple modalities, including conditioned medium, extracellular vesicles (EVs) and engineered delivery systems. Adipose-derived stem cells (ADSCs) conditioned medium restore dermal homeostasis by inhibiting UVB-induced senescence, enhancing collagen I/III and elastin synthesis, and suppressing MMPs (Guo et al, 2020). Clinical trials using ADSC-conditioned medium have demonstrated significant improvements in wrinkles, elasticity, pigmentation, and skin brightness (Putri et al, 2024; Wang et al, 2017c). Beyond the conditioned medium, ADSC-derived EVs rejuvenate dermal fibroblasts by restoring TGF-β1/TIMP-1 signaling and promoting ECM deposition, while suppressing UVB-induced overexpression of MMPs (Choi et al, 2019). Molecularly enhanced EVs, such as miR-1246-overexpressing vesicles, activate TGF-β/Smad signaling and inhibit MAPK/AP-1 and NF-κB pathways to prevent photoaging, while red-light-stimulated nanovesicles improve fibroblast migration and wound healing (Gao et al, 2023; Hyun et al, 2024). Engineered EV-based hydrogels and dermal fillers prolong collagen deposition and duration of therapeutic efficacy (You et al, 2022; You et al, 2024). In preclinical animal studies, ADSC-EVs or exosomes enhance dermal thickness, hair follicle regeneration, and angiogenesis (Li et al, 2022; Liang et al, 2020; Qin et al, 2021; Syromiatnikova et al, 2020). Clinical trials further confirm that ADSC-derived EVs improve wrinkles, elasticity, hydration, pigmentation, and collagen content in facial skin (Charles-de-Sá et al, 2020; Estupiñan et al, 2025; Park et al, 2023a).

Notably, other sources of mesenchymal stem cells (MSCs), including umbilical cord, placental, and hair follicle-derived MSCs, exhibit similar paracrine anti-aging effects. For example, conditioned medium from human umbilical cord-derived MSCs enhances skin brightness, reduces pigmentation and wrinkles, and improves elasticity (Liang et al, 2022b). Umbilical cord MSC-derived EVs protect human keratinocytes from UVB-induced photoaging by increasing proliferation, collagen I expression, and reducing MMP-1 expression (Liu et al, 2021a). Placental MSC-derived EVs delivered via chitosan hydrogel rejuvenate aged dermal fibroblasts by promoting proliferation, ECM synthesis, and suppressing SASP factors in vivo (Zhao et al, 2021b). Moreover, MSC interventions restore follicular β-catenin signaling, promote hair follicle regeneration, and reduce local inflammation (Deng et al, 2021; Yan et al, 2024). Systemic infusion of senescence-resistant mesenchymal progenitor cells in primates reprograms the skin transcriptomic clock by over five years, demonstrating a translational route for systemic skin rejuvenation (Lei et al, 2025). Collectively, stem cell-based therapies remodel aged dermal architecture through anti-inflammatory, and ECM-regenerative mechanisms, positioning engineered EVs and senescence-resistant MSCs as the next generation of skin rejuvenation modalities.

#### ECM remodeling

The dermal ECM serves as the structural and biomechanical foundation of youthful skin, supporting fibroblast function, elasticity, and intercellular signaling (Quan et al, 2013). With aging, aberrant collagen crosslinking, loss of matrix organization, and altered mechanotransduction perpetuate stiffness and cellular senescence (Fisher et al, 2009; Kamml et al, 2023; Rebehn et al, 2023). Strategies that actively remodel the ECM have emerged as promising approaches for skin rejuvenation.

Recent advances highlight matrix-targeted enzymatic modulation as a direct means to restore tissue pliability. Pharmacological inhibition of lysyl oxidases (LOXs), key enzymes catalyzing collagen crosslinking, has been shown to attenuate fibrosis and improve matrix architecture. Topical application of LOX inhibitors PXS-4787 ameliorated collagen deposition and crosslinking in murine and porcine skin scar models without compromising tensile strength (Chaudhari et al, 2022). A randomized, double-blind phase I trial of the LOX inhibitor PXS-6302 further demonstrated reduced LOX activity and increased microvessel density in mature scars (Morellini et al, 2025). These findings highlight the ECM-remodeling capacity of LOX inhibitors in skin fibrosis and scars, supporting their potential translational application for dermal ECM rejuvenation during skin aging.

Complementarily, biologically active scaffolds such as decellularized adipose matrix (DAM) rejuvenate photoaged skin by remodeling the immune–fibroblast axis. DAM injection promoted M2 macrophage polarization, thereby enhancing fibroblast-mediated ECM synthesis and increasing dermal thickness and collagen density in photoaged murine skin (Zhou et al, 2025a). Beyond exogenous interventions, endogenous remodeling stimuli also exert rejuvenative effects: resistance training was shown to enhance dermal ECM gene expression, improve elasticity, and thicken the dermis in middle-aged individuals (Nishikori et al, 2023). Together, these findings demonstrate that ECM remodeling can restore structural youthfulness and resilience—positioning ECM remodeling as a central pillar of skin rejuvenation.

### Systemic regulation

Skin aging is a multifactorial process influenced not only by local cellular and molecular mechanisms but also by systemic regulatory axes that integrate signals from distant organs and environmental exposures. Among these systemic influences, the microbiome and endocrine pathways have emerged as critical modulators of skin integrity, immune homeostasis, and aging phenotypes.

#### Microbiome modulation

Oral administration of specific probiotics and prebiotics has been shown to reinforce skin barrier and mitigate photoaging through the gut–skin axis. For instance, daily supplementation with probiotic strain *Lactobacillus plantarum* HY7714 improved skin hydration and attenuated photoaging-associated phenotypes, including wrinkles and loss of elasticity, in middle-aged adults (Lee et al, 2015). Similarly, daily intake of the prebiotic galacto-oligosaccharides (GOS) in combination with the probiotic *Bifidobacterium breve* reduced circulating phenolic toxins and improved skin barrier function in adult women, alleviating skin dryness and keratinization abnormalities (Kano et al, 2013). Proof‑of‑concept studies have further demonstrated that fecal microbiota transplantation (FMT) from young mice to aged mice can reverse key signs of skin aging, including increased stratum corneum thickness, enhanced collagen content, and promoted epidermal cell differentiation. Metabolomic analyses indicated that FMT elevated levels of tryptophan and its microbiota-derived metabolites, such as indole-3-lactic acid, suggesting that interventions targeting these metabolites may have potential to ameliorate age-related skin changes (Yu et al, 2024a).

#### Hormone regulation

Hormonal changes are a central driver of skin aging, influencing dermal structure, epidermal renewal, and barrier function. Estrogen replacement therapy have been shown to increase skin elasticity and collagen content, partially restore epidermal and dermal thickness, improve skin hydration, and reduce the severity of wrinkles in menopausal women, highlighting the critical role of estrogen signaling in maintaining skin integrity (Pivazyan et al, 2023). Androgens, including dehydroepiandrosterone (DHEA), testosterone, and dihydrotestosterone, also have great impact on skin aging. Topical or systemic androgen supplementation has been shown to partially reverse these changes: DHEA increases dermal procollagen I/III expression, enhances sebum production, and reduces MMP-1 activity, while combined estrogen–testosterone therapy increases type III collagen content in postmenopausal women (Brincat et al, 1983; El-Alfy et al, 2010; Nouveau et al, 2008; Savvas et al, 1993; Shin et al, 2005).

In addition to sex hormones, systemic anabolic hormones also influence skin aging. Clinical studies in GH‑deficient adults have shown that recombinant human GH therapy stimulates collagen type I synthesis, leading to increased dermal thickness (Kann et al, 1996). Similarly, experimental studies in aged mice show that plasmid-mediated delivery of growth hormone–releasing hormone elevates GH and IGF‑1 levels, enhances collagen synthesis, and increases dermal and epidermal thickness, while improving skin hydration and structural integrity (Ye et al, 2021). Glucocorticoid signaling also contributes to age-related skin changes. The enzyme 11β‑hydroxysteroid dehydrogenase type 1 (11β‑HSD1), which activates local glucocorticoids, increases with age in mouse skin. Pharmacological inhibition of 11β‑HSD1 enhances dermal thickness and collagen content in mice and promotes dermal fibroblast proliferation in vitro, suggesting that modulating local glucocorticoid metabolism may reverse dermal atrophy and collagen loss observed in aged or glucocorticoid-treated skin (Terao et al, 2014).

## Conclusion

Skin aging represents a visible manifestation of a coordinated multiscale ecosystem decline, arising from complex interactions among cellular senescence, metabolic dysregulation, epigenetic drift, and microenvironmental remodeling (An et al, 2025; Jankowski et al, 2025; Lee et al, 2021b; Martic et al, 2023; Zou et al, 2021). This review proposes that skin aging is not an irreversible accumulation of stochastic damage but a systemic shift in tissue homeostasis. Advances in multiscale assessment have transformed our ability to quantify this shift, positioning molecular and phenotypic aging clocks as integrated systems readouts that connect cellular alterations to functional outcomes. Crucially, emerging rejuvenation strategies—including transcriptional, metabolic, and microenvironmental interventions—demonstrate that the hallmarks of skin aging are reversible through multi-level rejuvenation of the tissue’s functional landscape, highlighting the skin’s inherent plasticity as a primary therapeutic target.

Future efforts should transition toward precision multi-level rejuvenation, integrating multiscale aging metrics with personalized interventions. By leveraging AI-driven analytics to combine molecular clocks, microbiome profiling, and high-resolution imaging, the dynamic monitoring and optimization of ecosystem restoration become feasible. Furthermore, the systemic impact of skin aging—such as the contribution of senescent cell burden to peripheral organ function and cognition, as well as interactions with the gut microbiome through the skin-gut axis—underscores the broader physiological benefits of skin-targeted therapies (Franco et al, 2025). Ultimately, viewing the skin as a controllable and restorative ecosystem paves the way for precision regenerative strategies that restore not only youthful skin but also systemic tissue resilience and organismal healthspan.

## Supplementary information

Peer Review File
