# Supplementary material for: Skin aging: mechanisms, evaluation, and rejuvenation
Source: EMBO J. 2026 May 21;45(14):4792–819. doi: 10.1038/s44318-026-00810-3 (PMC13373196; doi:10.1038/s44318-026-00810-3)
Supplement: Supplementary file 1 — Peer Review File [file 44318_2026_810_MOESM1_ESM.pdf]

THE EMBO JOURNAL

Skin Aging: Mechanisms, Evaluation, and Rejuvenation

Runhan Li, Jingyun Zhang, Kehang Mao, Dawei Meng, and Jing-Dong Han

Corresponding author: Jing-Dong Han (jackie.han@pku.edu.cn)

Review Timeline:

Submission Date:	27th Dec 25
Editorial Decision:	24th Feb 26
Revision Received:	10th Mar 26
Editorial Decision:	8th Apr 26
Revision Received:	9th Apr 26
Accepted:	14th Apr 26

Editor: Daniel Klimmeck

Transaction Report:

(Note: With the exception of the correction of typographical or spelling errors that could be a source of ambiguity, letters and reports are not edited. Depending on transfer agreements, referee reports obtained elsewhere may or may not be included in this compilation. Referee reports are anonymous unless the Referee chooses to sign their reports.)

Dear Dr Jacky Han,

Thank you again for sending us your review article for consideration, as well as for your patience with our feedback at this time.

We have now received dedicated input on your manuscript from two experts in skin aging specifically (Ref#1) and broader epithelial biology (Ref#2). As you will see, they appreciate the review and find it timely and worth publishing. They also provide constructive feedback on how to further improve and amend it by advancing the introduction and discussion, as well as clarifying a number of aspects. Further, they give advice on how to enhance the overall structure of the piece, and complement the literature context cited.

I hope you will find the comments helpful. I am sure that an amended version incorporating the suggestions made by the referees will be highly noted and appreciated. I would thus like to invite you to submit such a revised version using the link enclosed below.

Please let me know in case I can be of any help with this.

with
Best wishes to Beijing,

Daniel Klimmeck

Daniel Klimmeck, PhD
Senior Editor
The EMBO Journal

Further information is available in our Guide For Authors: https://www.embopress.org/page/journal/14602075/authorguide

----

Referee #1:

In this review the authors discuss the manifestations of cutaneous aging in the various cutaneous compartments and their effects, quantification of skin ageing and anti-aging therapeutics. This is a comprehensive review with some interesting discussion points. There are a number of potential improvements which I have been suggested below:

1. Intrinsic and extrinsic factors for ageing are referred to in general terms throughout the manuscript, with specific mention of UV radiation a number of times. This gives a somewhat unbalanced feel to what contributes to skin ageing. It would be good early on to specifically mention the different aspects of intrinsic ageing and the cutaneous exposome that contribute to cutaneous senescence and ageing (smoking, pollution etc). The various types of intrinsic and extrinsic factors for ageing should also be reflected in Figure 1.
2. In section 'Keratinocyte and stem cell dysfunction', final paragraph, the characteristics of aged skin being an inflammatory and proteolytic epidermal microenvironment is discussed. However one of the most characteristic aspects of aged skin in the epidermal compartment is epidermal thinning and its sequelae including altered barrier function, phenotypic changes etc. I would recommend including this.
3. Fibroblast senescence is perhaps the best characterised aspect of cellular senescence, and the authors summarise the known aspects in section 'Fibroblast senescence and ECM remodelling'. However this has been similarly reviewed elsewhere, and no new/novel/less well-reviewed concepts are introduced. An area gaining more traction is the changes in metabolism in senescent fibroblasts and its impact. It would be valuable to either include this or any novel concept/emerging research as relates to fibroblast senescence.
4. In section 'Immunosenescence and chronic inflammation', it is important to note that the cutaneous microenvironment in ageing skin causes immune cells to behave dysfunctionally, rather than there being a primary problem with the immune cells themselves, as demonstrated in this paper https://doi.org/10.1084/jem.20090896. There has further been a good discussion around utilising the microenvironment to boost immune responses in this paper DOI: 10.1038/s41573-024-01126-9
5. In section 'Evaluation of Skin Aging: How to quantify skin aging?' it would be worthwhile discussing the use of imaging technologies to detect advanced glycation end-products (AGEs) in the skin (https://doi.org/10.1111/exd.15065D), as this is a

currently-accessible aging assessment technology available to the public.
6. In section 'Evaluation of Skin Aging: How to quantify skin aging?', do the technologies discussed correlate to the standard biomarkers of ageing (e.g p16, p21, p53, TAF, SA-Beta-Gal etc)?
7. While comprehensive, the review does not have a common thread or strong narrative. Re-working the manuscript to contain a stronger narrative and novel conclusion would be beneficial.
8. Figure 2 will require some work on formatting to make it look cohesive, for example the labels 'Gut-Skin Axis', 'Aged Skin' and 'Hormonal Regulation' are not aligned correctly/are not on the same level; 'Aged Skin' is justified to the left rather than centrally and does not match with the other panels in the figure; consider including an outline box around the whole figure. Also clarify how 'Gut-Skin Axis', 'Aged Skin' and 'Hormonal Regulation' relate to intrinsic vs extrinsic causes for ageing e.g are these all intrinsic triggers for ageing?
9. There is some overlap with recent reviews: https://doi.org/10.3390/cosmetics12040144
https://doi.org/10.1016/j.archger.2025.105995

Referee #2:

This is a very impressive tour-de-force reviewing the multitude of factors affecting skin aging and the possible therapies targeting these factors. The review is well-written and highly informative. It considered both cell-intrinsic mechanisms as well as those systemic and related to the microenvironment, cellular-crosstalk, and even microbiota. In particular, the notion that skin aging is both influenced and influences systemic body aging is very interesting and worth considering, adding great value to the review. While I am not an expert in aging, I think the review is highly informative, interesting, and accessible to a broad audience.
I do have some minor suggestions for consideration to improve fairness along with the quality & accuracy of the review.
(1) The authors use aging studies that compare changes in certain parameters with homeostasis, but to be fair the original study describing the specific phenomenon in homeostasis, should also be cited. For example, the authors discuss the Slc1a3/Dlx1 epidermal stem cell populations and how they change in aging, but it'd be only fair to cite the original papers Sada et al, 2016 (Nat Cell Bio) and Ghuwalewala et al 2024 (Development) that defined and characterized the populations during homeostasis and although not focused on aging they followed their behavior throughout most of the mouse life.

(2) The skin morphology depicted in figures 1&2 is not representative of human skin. Most human skin samples show undulation of the basal layer (rete ridges and inter-ridges) but the top surface is relatively flat. This makes the rete ridge progenitors (Slc1a3+) more deeply embedded in the dermis than the inter-ridge progenitors, and likely their progenitors are more protected from the environment. Ghuwalewala et al (2022, EMBO J) provides evidence that more embedded Slc1a3+ are more protected from UV and given the intense focus of the review on UV exposure, this aspect of skin organization may be interesting to consider along with Slc1a3/Dlx1 populations that were already discussed.

(3) The function of telomeres and telomerase in skin aging have been studied in mice via both deletion of TERC over five mouse generations and overexpression of TERT. Both o modifications affect skin stem cells self-renewal and demonstrate contribution to skin aging. Diskeratosis congenita, a telomere length human genetic disease, is mentioned very briefly, but not as a clear illustration of how some aspects of skin aging (hair greying, depigmentation, hair loss) can be linked to telomere length in human.

(4) An emerging factor in aging is loss of silencing at transposable elements, which can occur due to increase inflammation or loss of chromatin and youthful epigenetic patterns. While I am not sure how much is known exactly in skin about this, it is a general factor considered in aging. A recent paper from the Ge lab (Cell 2025) showed that loss of a single histone methylase can activate transposons in skin, affecting hair follicle stem cell activation patterns. Although they have not been directly linked this to aging, evidence exists that transposons may be aberrantly activated in inflammatory skin disease, such as psoriasis. The authors consider inflammation a lot in the review, it may be interesting to link it with transposons even if skin currently lacks evidence for the implication of these aging factors.

(5) Some citations are incomplete - for example it would just say the title of the journal - Nature - but no volume and number and page info. Please double check that all citations are complete and conforming with the journal's format.

(6) Figures 1&2 are cited in the intro and that is a nice overview to begin with, but it is not clear where all the things in the figure panels are coming from. It would be nice to cite these figures again in text as the data is discussed in more detail.

Overall, a very great job.

Point-by-Point Response to Reviewers

Please note that the reviewer's concerns are listed one by one below (black characters), followed by our responses (blue characters) and, when relevant, the corresponding changes in the text (red characters).

Reviewer #1 (Remarks to the Author):

In this review the authors discuss the manifestations of cutaneous aging in the various cutaneous compartments and their effects, quantification of skin ageing and anti-aging therapeutics. This is a comprehensive review with some interesting discussion points. There are a number of potential improvements which I have been suggested below:

We would like to thank the reviewer for their thoughtful and constructive comments on our manuscript. These suggestions have significantly helped us to improve the balance, novelty, and clarity of this review. We have addressed each point carefully, as detailed below.

1. Intrinsic and extrinsic factors for ageing are referred to in general terms throughout the manuscript, with specific mention of UV radiation a number of times. This gives a somewhat unbalanced feel to what contributes to skin ageing. It would be good early on to specifically mention the different aspects of intrinsic ageing and the cutaneous exposome that contribute to cutaneous senescence and ageing (smoking, pollution etc). The various types of intrinsic and extrinsic factors for ageing should also be reflected in Figure 1.

We thank the reviewer for this important suggestion. We agree that the previous version over-emphasized UV exposure relative to other contributors to skin aging. In the revised manuscript, we strengthened the conceptual framework early in the review by explicitly defining two synergistic axes that drive cutaneous aging: intrinsic aging mechanisms and the cutaneous exposome (Page 2, Lines 69-89) .

Page 2, Lines 69-89: "Conceptually, this process is driven by the convergence of two distinct yet synergistic axes: intrinsic aging mechanisms and the cutaneous exposome (Naharro-Rodriguez *et al*, 2025a). Intrinsic factors—such as stem cell exhaustion, genomic instability, epigenetic drift, and mitochondrial dysfunction—interact with a broad spectrum of extrinsic stressors. Importantly, the cutaneous exposome extends beyond well-characterized ultraviolet (UV) radiation to include particulate air pollution, tobacco smoke, and nutritional or climatic stressors (Fig. 1). Collectively, these forces disrupt intercellular communication and tissue homeostasis. A central consequence of this convergence is progressive remodeling of the cutaneous microenvironment."

To reflect this framework visually, Figure 1 has been revised to explicitly depict both axes: (i) an expanded cutaneous exposome module (air pollution, UV radiation, tobacco smoke, nutrition, climate) and (ii) an intrinsic aging module (stem cell exhaustion, genomic instability, mitochondrial dysfunction), with convergent arrows

indicating joint impacts on the skin ecosystem.

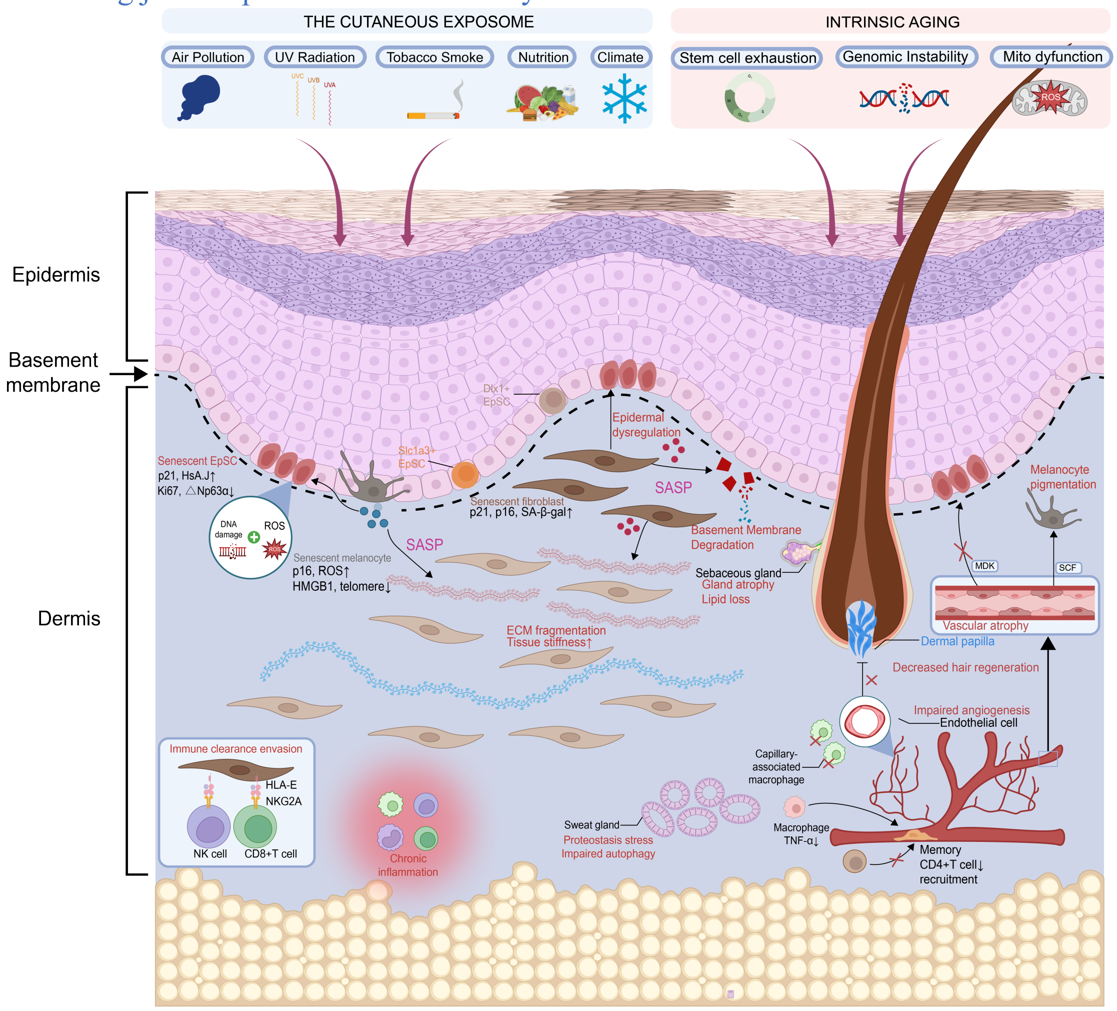

Figure 1. Systemic deterioration of the skin ecosystem during aging. Schematic cross-section of aged human skin illustrating how intrinsic aging mechanisms (stem cell exhaustion, genomic instability, and mitochondrial dysfunction) converge with the cutaneous exposome (air pollution, ultraviolet radiation, tobacco smoke, nutritional factors, and climatic stressors) to disrupt intercellular communication and tissue homeostasis (top). These combined inputs promote multicellular senescence and microenvironmental remodeling—characterized by SASP signaling, basement membrane degradation, ECM fragmentation/stiffening, chronic inflammation, vascular dysfunction, and impaired appendageal regeneration—culminating in tissue-level functional decline across the epidermis, dermis, and skin appendages.

2. In section 'Keratinocyte and stem cell dysfunction', final paragraph, the characteristics of aged skin being an inflammatory and proteolytic epidermal microenvironment is discussed. However one of the most characteristic aspects of aged skin in the epidermal compartment is epidermal thinning and its sequelae including altered barrier function, phenotypic changes etc. I would recommend including this.

We thank the reviewer for highlighting this key hallmark of epidermal aging. We agree that epidermal thinning and barrier impairment represent central tissue-level

manifestations of epidermal aging and should be explicitly discussed. Accordingly, we expanded the the "Keratinocyte and stem cell dysfunction" subsection to include epidermal thinning and its major consequences (Page 4, Lines 143-152).

Page 4, Lines 143-152: "These intrinsic and extrinsic processes converge at the tissue level: aged human epidermis commonly exhibits epidermal thinning and progressive flattening of the dermal–epidermal junction (DEJ) with loss of rete ridges, reducing the exchange surface area and contributing to mechanical fragility and delayed repair (Branchet *et al*, 1990; Rittié & Fisher, 2015; Roig-Rosello & Rousselle, 2020). Functionally, even when baseline permeability measures (such as transepidermal water loss, TEWL) appear modestly changed, aged skin is more readily perturbed and shows delayed barrier recovery after disruption, consistent with compromised regenerative and lipid/lamellar body programs (Ghadially *et al*, 1995)."

We integrated this content to connect cellular-level keratinocyte/EpSC dysfunction with clinically relevant tissue phenotypes, improving the completeness and translational relevance of this section.

3. Fibroblast senescence is perhaps the best characterised aspect of cellular senescence, and the authors summarise the known aspects in section 'Fibroblast senescence and ECM remodelling'. However this has been similarly reviewed elsewhere, and no new/novel/less well-reviewed concepts are introduced. An area gaining more traction is the changes in metabolism in senescent fibroblasts and its impact. It would be valuable to either include this or any novel concept/emerging research as relates to fibroblast senescence.

We thank the reviewer for this helpful suggestion and agree that classical fibroblast senescence and ECM remodeling have been comprehensively reviewed. To strengthen novelty and provide a more forward-looking perspective, we expanded this section by adding a new paragraph highlighting metabolic reprogramming as an emerging upstream determinant of senescent fibroblast phenotypes and matrix remodeling (Page 7, Lines 293-309).

Page 7, Lines 293-309: "Beyond classical DNA damage and ROS pathways, accumulating evidence indicates that metabolic reprogramming is a key upstream determinant of senescent fibroblast phenotypes and matrix remodeling. Metabolomic profiling of senescent human fibroblasts revealed increased glycolysis and pentose phosphate pathway activity, together with a characteristic extracellular "senescence metabolome" enriched for metabolites such as citrate and oxidative-stress–related molecules, supporting a functional link between metabolic state and senescence-associated tissue phenotypes (James *et al*, 2015; James *et al*, 2018). Nutrient-sensing circuitry links this metabolic state to inflammatory secretomes: mTORC1 promotes IL-1α translation to sustain NF-κB–driven SASP programs, and rapamycin selectively blunts this pro-inflammatory output (Laberge *et al*, 2015). In parallel, NAD^+ salvage metabolism (HMGA–NAMPT–NAD^+ axis) governs the pro-inflammatory SASP independently of growth arrest via AMPK–p53–p38–NF-κB signaling, emphasizing that "senescence metabolism" actively shapes secretory and matrix-degrading

programs (Nacarelli *et al*, 2019). Consistent with a bioenergetic "dial" on SASP composition, mitochondrial dysfunction–associated senescence (MiDAS) features a lower NAD^+/NADH ratio and a modified SASP that lacks the IL-1–dependent inflammatory arm (Wiley *et al*, 2016). "

Together, these additions provide a more forward-looking, mechanism-linked view of fibroblast senescence in dermal aging.

4. In section 'Immunosenescence and chronic inflammation', it is important to note that the cutaneous microenvironment in ageing skin causes immune cells to behave dysfunctionally, rather than there being a primary problem with the immune cells themselves, as demonstrated in this paper https://doi.org/10.1084/jem.20090896. There has further been a good discussion around utilising the microenvironment to boost immune responses in this paper DOI: 10.1038/s41573-024-01126-9

We thank the reviewer for this important conceptual correction and for the suggested references. We agree that immune dysfunction in aged skin should be framed as a tissue-level phenomenon resulting from both immune-cell intrinsic changes and microenvironment-imposed constraints. In the revised manuscript, we restructured this section to explicitly emphasize the role of the aged cutaneous niche in limiting immune surveillance, recruitment, and activation. In parallel, we expanded the discussion of translational implications by incorporating the recent perspective that targeting the tissue environment (e.g., senescence–inflammation–immune interactions) may enhance immune function during aging (Page 9, Lines 369-399).

Page 9, Lines 369-399: "T cell–mediated immunosurveillance in aged skin is likewise increasingly constrained by a suppressive tissue milieu. Although varicella-zoster virus (VZV)-specific $CD4^+$ T cells are enriched in human skin and remain functionally competent in older individuals, aged skin exhibits an increased local inhibitory tone, including higher $Foxp3^+$ regulatory T cells and elevated PD-1 expression on cutaneous $CD4^+$ T cells, which may restrain effective immune responses in situ (Vukmanovic-Stejic *et al*, 2015). While intrinsic factors such as reduced autophagic capacity (He *et al*, 2024) may contribute to immune dysfunction, accumulating evidence indicates that age-related impairment of cutaneous recall responses is often rate-limited by the tissue microenvironment rather than a primary defect in memory T cell skin-homing or migratory potential. Specifically, defective activation of dermal blood vessels in older individuals—driven by reduced macrophage-derived TNF-α—restricts memory $CD4^+$ T cell entry into antigen-challenged skin, and macrophage TNF-α production can be at least partially restored upon innate (TLR) stimulation, underscoring a reversible microenvironmental bottleneck (Agius *et al*, 2009) (Fig. 1). This microenvironmental suppression is further exacerbated by extrinsic stressors, which promotes expansion of regulatory T cells and perturbs local immune homeostasis (Yamazaki *et al*, 2014). Collectively, these changes bias aged skin toward a state of chronic, low-grade inflammation with impaired immune surveillance and delayed repair.

Consequently, there is growing interest in utilizing the tissue microenvironment to

boost immune responses in aged skin. Reflecting this paradigm, recent therapeutic perspectives emphasize breaking the senescence–inflammageing–immune dysfunction cycle by targeting interactions between the altered tissue environment and immune cells (for example, senolytics and inflammation-modulating “senomorphics”), thereby restoring tissue immune surveillance and improving immune responsiveness during ageing (Bracken *et al*, 2025; Kirkland & Tchkonia, 2020; Wang & Nakanishi, 2025). Consistent with the concept that the aged niche can be therapeutically reprogrammed, early sterile inflammation and p38 MAPK–linked cytokine production in older skin are associated with attenuated VZV challenge responses, whereas short-term p38 inhibition (losmapimod) enhances antigen-specific cutaneous immunity in older individuals (Vukmanovic-Stejic *et al*, 2018).”

Finally, we ensured this logic is reflected in Figure 1 by annotating the macrophage–vascular–T cell recruitment axis as an example of microenvironmental control over immune behavior.

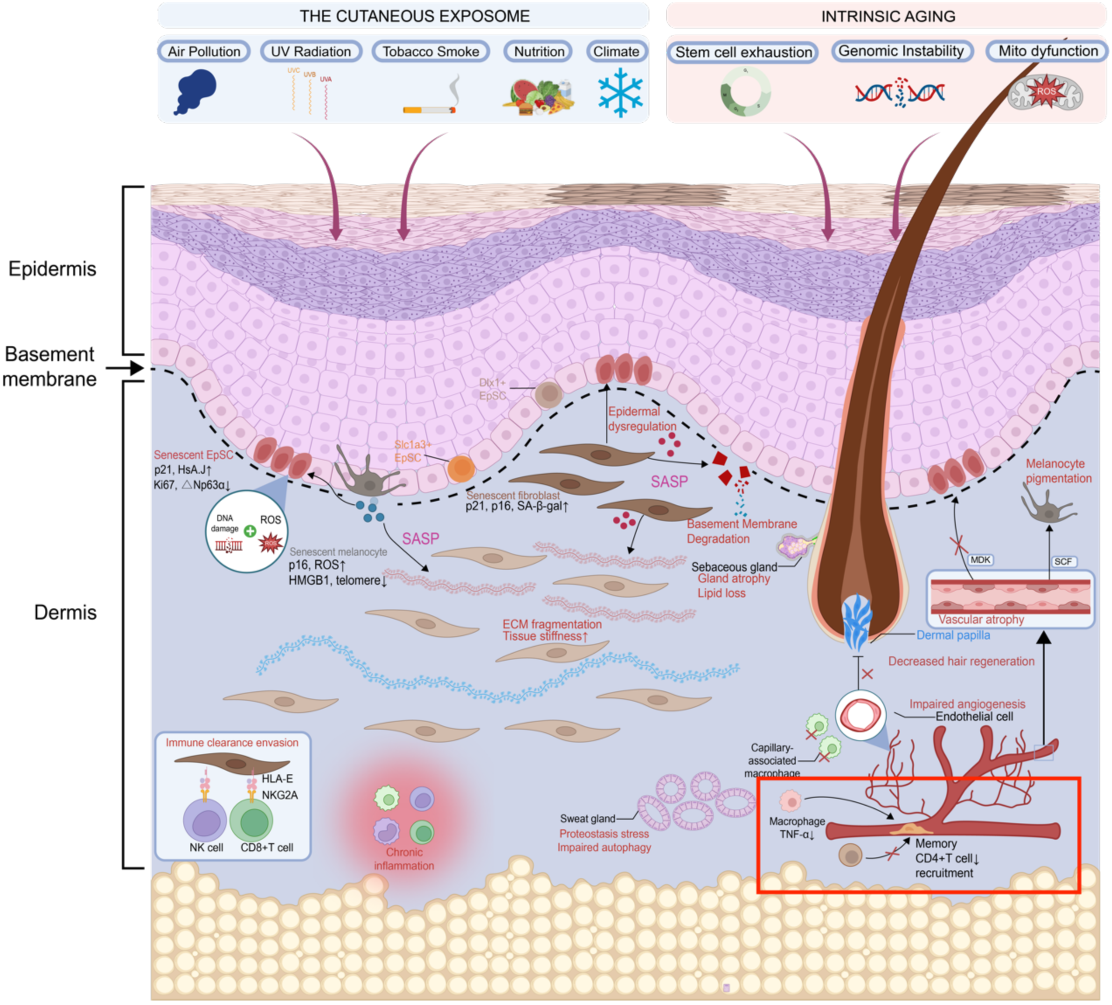

Figure 1. Systemic deterioration of the skin ecosystem during aging. Schematic cross-section of aged human skin illustrating how intrinsic aging mechanisms (stem cell exhaustion, genomic instability, and mitochondrial dysfunction) converge with the cutaneous exposome (air pollution, ultraviolet radiation, tobacco smoke, nutritional factors, and climatic stressors) to disrupt intercellular communication and tissue homeostasis (top). These combined inputs promote multicellular senescence and

microenvironmental remodeling—characterized by SASP signaling, basement membrane degradation, ECM fragmentation/stiffening, chronic inflammation, vascular dysfunction, and impaired appendageal regeneration—culminating in tissue-level functional decline across the epidermis, dermis, and skin appendages.

5. In section 'Evaluation of Skin Aging: How to quantify skin aging?' it would be worthwhile discussing the use of imaging technologies to detect advanced glycation end-products (AGEs) in the skin (https://doi.org/10.1111/exd.15065D), as this is a currently-accessible aging assessment technology available to the public.

We sincerely thank the reviewer for this insightful suggestion. We agree that advanced glycation end-products (AGEs) represent a critical molecular biomarker of skin aging, linking metabolic dysfunction to structural deterioration. The accumulation of AGEs leads to aberrant collagen crosslinking and increased matrix stiffness, which are fundamental to the aging phenotype. In accordance with your suggestion, we have now integrated a discussion on AGEs-based assessment in the revised section "Evaluation of Skin Aging: How to quantify skin aging?" (Page 13, Lines 650-660).

Page 13, Lines 650-660: "Beyond direct morphological changes, structural integrity is further compromised by the accumulation of advanced glycation end-products (AGEs) in the dermal matrix (Odetti et al, 1992). These stable products, formed via non-enzymatic glycation of long-lived proteins like collagen, lead to irreversible cross-linking that stiffens the skin and contributes to sallow discoloration (Corstjens et al, 2008; Fessel et al, 2014). Notably, the development of imaging technologies, such as skin autofluorescence (SAF), has enabled the non-invasive quantification of tissue AGEs in vivo (Birukov et al, 2021). As an accessible aging assessment technology, SAF serves as a biophysical proxy for cumulative metabolic and structural damage, bridging the gap between molecular glycation and visible phenotype (Koetsier et al, 2010; Majchrzak et al, 2022; Waqas et al, 2022)."

In addition, this assessment approach has now been incorporated into Table 1 to provide a clearer overview of current skin aging evaluation strategies.

6. In section 'Evaluation of Skin Aging: How to quantify skin aging?', do the technologies discussed correlate to the standard biomarkers of ageing (e.g p16, p21, p53, TAF, SA-Beta-Gal etc)?

We sincerely appreciate the reviewer's insightful suggestion. We agree that classical cellular senescence biomarkers represent widely accepted molecular indicators of aging and senescence, and that linking evaluation technologies to these biomarkers helps clarify the biological basis underlying quantitative skin-aging assessment.

First, for traditional methods, histological approaches can directly quantify classical senescence markers when combined with appropriate staining or immunohistochemical techniques, including p16, p21, p53 signaling activity, and SA-β-gal staining. Several fluid biomarkers described in the manuscript are also linked to cellular senescence. In particular, SASP factors (e.g., IL-8, IL-1β, and MMPs) represent

downstream products of senescent cells and are frequently regulated by the p53/p21 and p16^{INK4a} /Rb pathways. Markers of oxidative DNA damage, such as 8-OHdG, further reflect genomic stress that contributes to the induction of cellular senescence.

Second, for various aging clocks, studies examining their direct correlation with p16, p21, or SA-β-Gal remain limited. However, several studies suggest that these models capture biological processes associated with senescence-related pathways. For example, the DNA methylation clock VisAgeX identified enrichment of pathways linked to skin aging, including estrogen response, UV-responsive genes, hypoxia response, and epithelial–mesenchymal transition (Bienkowska *et al*, 2023). Similarly, a transcriptomic aging clocks based on hallmark gene sets highlighted p53 and TNFα/NFκB signaling pathways as major drivers associated with epidermal aging (Holzscheck *et al*, 2021). In addition, the phenotype clock FaceAge showed a significant association with the aging-related gene CDK6, suggesting that image-based aging predictors may partially reflect molecular pathways involved in aging. We have now expanded our discussion of these mechanistic intersections in the revised manuscript:

DNA methylation clock VisAgeX (Page 14, Lines 718-723): "More recently, the VisAgeX clock, which is based on large methylation regions, predicts visual skin aging with a MAE of 4-6 years. In addition to its predictive performance, pathway enrichment analysis further identified several biological processes linked to skin aging, including estrogen response, UV-responsive genes, hypoxia response, and epithelial–mesenchymal transition (Bienkowska *et al.*, 2023)."

Transcriptomic aging clock (Page 15, Lines 747-751): "Notably, these pathways are closely linked to canonical cellular senescence regulators, as p53 signaling is a key mediator of p21-dependent cell-cycle arrest, while NFκB signaling drives the SASP. This suggests that transcriptomic aging clocks may partially capture molecular programs associated with senescence, even though they do not directly measure classical senescence biomarkers such as p16, p21, or SA-β-Gal."

Phenotype clock FaceAge (Page 17, Lines 804-810): "Notably, the FaceAge model estimates biological age from facial images and demonstrates independent prognostic value across multiple cancer types, with patients appearing on average 4.79 years older than their chronological age. Importantly, FaceAge showed a significant association with the aging-related gene CDK6, suggesting that image-based aging predictors may partially reflect molecular pathways involved in biological aging and cellular senescence (Bontempi *et al*, 2025)."

Taken together, the technologies described above primarily capture downstream phenotypic or pathway-level manifestations of aging processes that are mechanistically linked to cellular senescence, rather than directly quantifying canonical senescence biomarkers.

7. While comprehensive, the review does not have a common thread or strong narrative. Re-working the manuscript to contain a stronger narrative and novel conclusion would be beneficial.

We thank the reviewer for this valuable high-level comment. We agree that strengthening the manuscript's narrative thread substantially improves readability and conceptual impact.

We have refined the manuscript to center on a unified conceptual framework: **Skin aging as a multi-scale ecosystem decline and rejuvenation as systems reprogramming.** This framework provides a robust narrative thread that seamlessly connects the three core pillars of our review:

a. Introduction: we have revised the Introduction to articulate the overarching structure and narrative logic of the review more explicitly. The manuscript has been revised as follows:

Page 2, Lines 56-65: "This review first explores the multi-layered cellular and molecular mechanisms of aging across the epidermis, dermis, and appendages, highlighting how a breakdown in inter-compartmental communication undermines the skin ecosystem's integrity. We then examine current approaches for assessing skin aging at multiple scales, from molecular signatures to tissue-level phenotypes, framing these aging clocks as integrated systems readouts of the skin's biological state. Finally, we highlight recent advances in rejuvenation strategies to reverse age-related changes, encompassing transcriptional, metabolic, microenvironmental, microbial, and hormonal interventions that aim to reprogram and restore youthful cellular function and systemic tissue resilience."

b. Mechanisms (Systemic deterioration of the skin ecosystem: why does the skin age?): We have re-structured the narrative to reflect ecosystemic decline. We further emphasize the breakdown of inter-compartmental communication. We have integrated recent evidence showing how niche stiffening and 'cellular identity drift' function as systemic drivers that lock the skin ecosystem into a self-reinforcing senescent state.

Page 3, Lines 94-98: "Therefore, skin aging is not merely the sum of individual cellular changes but the outcome of interconnected failures spanning molecular, tissue, and systemic levels. Understanding this ecosystemic collapse provides the necessary framework for developing systems reprogramming strategies aimed at restoring youthful functional integrity."

Disrupted crosstalk across epidermis, dermis and appendages Part (Page 9, Lines 401-517) : "Skin aging is fundamentally characterized by a progressive collapse of the multi-scale ecosystem, driven by a breakdown in multicellular crosstalk that couples immune evasion, inflammatory signaling, aberrant mechanotransduction, and niche failure across dermal and epidermal compartments. The accumulation of senescent cells serves as a primary catalyst for this decline, fostering a pro-inflammatory and structurally compromised niche that propagates aging signals through the tissue (Furman et al, 2025).

Within this failing ecosystem, senescent fibroblasts in the dermis actively evade immune clearance by NK and CD8+ T cells through HLA-E upregulatio (Pereira et al, 2019) (Fig. 1). These cells further destabilize the microenvironment by secreting CCL2 to recruit inflammatory monocytes, which release prostaglandin E_2 (PGE2) to suppress local T-cell memory and impair immune surveillance (Chambers et al, 2021).

Simultaneously, in the epidermis, senescent melanocytes establish a self-reinforcing senescence loop via IP-10/CXCR3 signaling. This axis also induces "bystander" senescence in neighboring keratinocytes and fibroblasts through paracrine signals that elevate ROS and DNA damage, contributing to epidermal atrophy (Victorelli et al., 2019).

The integrity of the dermal niche is further eroded by the synergy of vascular decline and mechanical remodeling. UV-irradiated endothelial cells secrete stem cell factor (SCF), promoting melanocyte hyperpigmentation (Kim et al, 2018). Concurrently, age-related vascular atrophy increases dermal stiffness, which activates the mechanosensitive channel Piezo1 in epidermal stem cells, driving their premature differentiation (Ichijo et al, 2022). This stiffening also disrupts a critical fibroblast-endothelial-epidermal metabolic retinoid axis (MDK-SDC4-RBP1), impairing epidermal self-renewal (Wu et al., 2025) (Fig. 1).

The impact of ecosystemic deterioration is also evident in skin appendages , where the aged dermal niche dictates stem cell behavior (Ge et al, 2020). Mechanistically, increased matrix stiffness silences key HFSCs genes, inhibiting their activation (Koester et al., 2021). Within this failing niche, dermal papilla-vascular communication weakens, while macrophage-derived cytokines signal via JAK-STAT pathways to enforce quiescence and further suppress regenerative activity (Wang et al, 2019; Zhou et al, 2025b) (Fig. 1). Collectively, this intricate network of dysfunctional signaling drives the systemic breakdown of skin structure and function, reinforcing the need to view skin aging as a holistic system failure."

c. Evaluation (Multiscale Assessment of the Skin Ecosystem: How to quantify skin aging?): For the evaluation section, we have re-titled it 'Multiscale Assessment of the Skin Ecosystem: How to quantify skin aging?'. We revised this section to better emphasize the multi-scale framework for assessing skin aging. The text now highlights the integration of aging clocks across different biological levels. We further clarify that these complementary measurements provide systems-level readouts of the skin ecosystem. The manuscript has been revised as follows:

Page 17, Lines 838-859: "Accurate skin aging clocks capture the biological state of the skin ecosystem across multiple scales, ranging from molecular signatures such as DNA methylation clocks, to cellular phenotypic clocks based on morphology and functional states, and ultimately to organism-level indicators including 3D facial phenotypes. Together, these complementary metrics create a comprehensive digital representation of the skin ecosystem, linking intracellular molecular hallmarks with macroscopic tissue and organismal features. Collectively, these approaches establish a largely non-invasive, multi-scale phenotyping framework that enables longitudinal and interventional assessment of human skin aging. Importantly, such measurements allow dynamic monitoring of how the system deviates from youthful homeostasis, thereby providing quantitative baselines to identify precise intervention points for rejuvenation and cellular reprogramming strategies. Future integration of these diverse data streams through AI and machine-learning frameworks may further enable personalized skin-aging profiles and adaptive monitoring of rejuvenation interventions."

d. Rejuvenation (Restoring the Skin Ecosystem: Strategies for Multi-level

Rejuvenation): For the rejuvenation section, we have re-titled it 'Multiscale Assessment of the Skin Ecosystem: How to quantify skin aging?'. We now explicitly state that skin aging represents a coordinated ecosystem decline, and therefore, rejuvenation requires a holistic reset of the tissue's molecular, cellular, and microenvironmental landscapes. This restructuring provides the strong, conceptual thread the reviewer requested, linking the multifaceted interventions into a singular, unified framework.

Page 18, Lines 862-873: "Skin aging reflects a coordinated decline of a multi-scale ecosystem, driven by transcriptomic dysregulation, epigenetic drift, metabolic decline, and microenvironmental imbalance, as well as systemic microbial and hormonal dysregulation (An *et al*, 2025; Jankowski *et al*, 2025; Lee *et al*, 2021; Martic *et al*, 2023; Zou *et al*, 2021). Rather than merely delaying deterioration, contemporary rejuvenation strategies aim to reverse these age-related alterations through systems reprogramming. This paradigm shift focuses on restoring youthful homeostatic equilibrium across molecular, cellular, and microenvironmental levels, while simultaneously recalibrating systemic axes to ensure tissue-wide resilience (Fig. 3). By framing rejuvenation as a holistic 'reset' of the skin system, we can integrate diverse interventions—from cellular rewiring to micro-environmental remodeling—into a unified therapeutic roadmap."

e. Conclusion: In conclusion, we further emphasize the conceptual framework: conceptual framework: 'Skin aging as a multi-scale ecosystem decline and rejuvenation as systems reprogramming'. By shifting the narrative from a summary of isolated mechanisms to a holistic view of ecosystem restoration, we provide a more cohesive and forward-looking perspective.

Page 26, Lines 1233-1254: "This review proposes that skin aging is not an irreversible accumulation of stochastic damage but a systemic shift in tissue homeostasis. Advances in multiscale assessment have transformed our ability to quantify this shift, positioning molecular and phenotypic aging clocks as integrated systems readouts that connect cellular alterations to functional outcomes. Crucially, emerging rejuvenation strategies—including transcriptional, metabolic, and microenvironmental interventions—demonstrate that the hallmarks of skin aging are reversible through multi-level rejuvenation of the tissue's functional landscape, highlighting the skin's inherent plasticity as a primary therapeutic target.

Future efforts should transition toward precision multi-level rejuvenation, integrating multi-scale aging metrics with personalized interventions. By leveraging AI-driven analytics to combine molecular clocks, microbiome profiling, and high-resolution imaging, the dynamic monitoring and optimization of ecosystem restoration become feasible. Furthermore, the systemic impact of skin aging—such as the contribution of senescent cell burden to peripheral organ function and cognition, as well as interactions with the gut microbiome through the skin-gut axis—underscores the broader physiological benefits of skin-targeted therapies (Franco et al, 2025). Ultimately, viewing the skin as a controllable and restorative ecosystem paves the way for precision regenerative strategies that restore not only youthful skin but also systemic tissue resilience and organismal healthspan."

8. Figure 2 will require some work on formatting to make it look cohesive, for example the labels 'Gut-Skin Axis', 'Aged Skin' and 'Hormonal Regulation' are not aligned correctly/are not on the same level; 'Aged Skin' is justified to the left rather than centrally and does not match with the other panels in the figure; consider including an outline box around the whole figure. Also clarify how 'Gut-Skin Axis', 'Aged Skin' and 'Hormonal Regulation' relate to intrinsic vs extrinsic causes for ageing e.g are these all intrinsic triggers for ageing?

We sincerely appreciate the reviewer's meticulous attention to the presentation and logical structure of Figure 2. We apologize for the previous formatting inconsistencies and the lack of clarity regarding the categorization of these factors, which we agree could lead to potential confusion. We have thoroughly redesigned the figure to ensure all labels are precisely aligned, "Aged Skin" is centered, and an outline box is added for improved cohesion. Furthermore, we have refined the underlying logic by clearly categorizing "Hormonal Regulation" as an intrinsic factor, while presenting the "Gut-Skin Axis" as a extrinsic mediator that bridges both extrinsic influences and intrinsic biological processes.

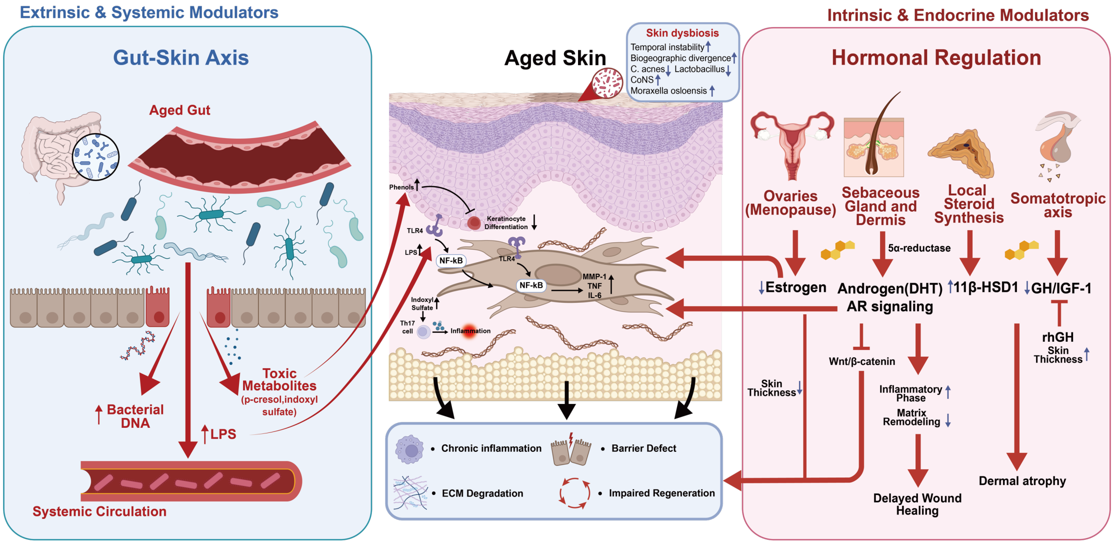

Figure 2. Converging gut–skin microbiome and endocrine mechanisms in skin aging. The figure is arranged as three framed panels to emphasize how extrinsic/systemic and intrinsic/endocrine modulators converge on shared tissue outcomes. Left (Extrinsic & systemic; Gut–Skin Axis): age-associated gut dysbiosis and barrier leakiness promote systemic dissemination of microbial products (e.g., lipopolysaccharide (LPS), bacterial DNA) and metabolites (e.g., p-cresol, indoxyl sulfate), which are strongly shaped by exposome inputs (diet, medications, smoking/pollutants). Middle (Aged skin): circulating cues activate pattern-recognition pathways (e.g., TLR4) in keratinocytes and fibroblasts, driving NF-κB–linked inflammation and matrix metalloproteinase activity (e.g., MMP-1), impairing differentiation and accelerating extracellular matrix (ECM) breakdown; skin dysbiosis may further amplify these responses. Right (Intrinsic & endocrine; Hormonal regulation): reduced estrogen and GH/IGF-1 weaken collagen/skin thickness and

regenerative capacity, while increased local androgen signaling (5α-reductase–dependent DHT/androgen receptor (AR)) antagonizes Wnt/β-catenin programs and delays wound healing; elevated 11β-HSD1 enhances glucocorticoid activation and dermal atrophy. Together, these axes bridge intrinsic aging and exposome inputs to drive chronic inflammation, barrier defects, and impaired repair.

9. There is some overlap with recent reviews: https://doi.org/10.3390/cosmetics12040144
https://doi.org/10.1016/j.archger.2025.105995

We thank the reviewer for pointing out these two recent and excellent reviews. We have carefully studied both articles and would like to clarify the unique contributions and distinct focus of our manuscript, which sets it apart from these existing publications:

a. Distinction from Naharro-Rodriguez et al. (Naharro-Rodriguez *et al*, 2025b):

- Naharro-Rodriguez et al. primarily offer a comprehensive overview of clinical and histological changes and established dermatological therapies, such as retinoids, lasers, and fillers. While both reviews address mechanisms and therapies, Naharro-Rodriguez et al. categorize mechanisms by the traditional split of intrinsic and extrinsic factors.
- In contrast, **our review treats the skin as a multi-layered ecosystem, dissecting aging into the deterioration of distinct compartments and the complex crosstalk between them**. Furthermore, **our work emphasizes systemic regulators**, including the gut–skin axis and hormonal shifts, positioning skin aging not as a passive consequence but as an active participant in organismal aging. We present a novel synthesis of the systemic impact of skin aging, where aged skin acts as a regulator of distal organ function. This "systemic sentinel" perspective frames skin-targeted therapies as a means to enhance overall organismal healthspan, providing a broader physiological narrative compared to the localized focus of traditional dermatological reviews.
- Unlike Naharro-Rodriguez et al. focuses on established dermatological procedures, our manuscript highlights **next-generation reprogramming strategies**, such as TF-mediated and chemical partial reprogramming. These approaches aim to rejuvenate the aging system’s transcriptional and epigenetic landscape rather than merely delaying deterioration.

b. Distinction from Wang et al. (Wang *et al*, 2025):

- Wang et al. focused specifically on the validation and reliability of clinical grading scales.
- While traditional structural and functional assessments are summarized in our work, they represent only a small portion of our evaluation framework. **Our review places much greater emphasis on multimodal aging clocks**—including DNA methylation, transcriptomic, miRNA-based, and cellular morphology clocks—as integrated systems readouts of biological age. We provide an in-depth analysis of how these molecular and AI-driven metrics quantify the system's shift from youthful resilience to senescent fragility, a major focus not extensively covered by

Wang et al..

We have cited these suggested reviews in the corresponding sections of our revised manuscript to ensure that interested readers can refer to their specific clinical and methodological insights.

Page 2, Lines 69-71: "Conceptually, this process is driven by the convergence of two distinct yet synergistic axes: intrinsic aging mechanisms and the cutaneous exposome (Naharro-Rodriguez *et al.*, 2025b)."

Page 13, Lines 643-645: "Traditional assessment of skin aging encompasses several interrelated aspects, including structural integrity, functional performance, and fluid biomarkers (Wang *et al.*, 2025)."

Reviewer #2 (Remarks to the Author):

This is a very impressive tour-de-force reviewing the multitude of factors affecting skin aging and the possible therapies targeting these factors. The review is well-written and highly informative. It considered both cell-intrinsic mechanisms as well as those systemic and related to the microenvironment, cellular-crosstalk, and even microbiota. In particular, the notion that skin aging is both influenced and influences systemic body aging is very interesting and worth considering, adding great value to the review. While I am not an expert in aging, I think the review is highly informative, interesting, and accessible to a broad audience.

We sincerely thank the reviewer for the generous and thoughtful evaluation of our manuscript. We are particularly encouraged that the integrative framework—encompassing cell-intrinsic mechanisms, systemic influences, microenvironmental remodeling, intercellular crosstalk, and microbiota—was recognized as a strength of the review. We also appreciate the positive feedback regarding our discussion of the bidirectional relationship between skin aging and systemic organismal aging; our intention was to highlight the skin not merely as a passive target of aging processes, but as an active participant in shaping systemic physiology, and we are pleased that this perspective was viewed as adding conceptual value. Finally, we are grateful for the comment that the review is informative, interesting, and accessible to a broad audience, as we aimed to balance mechanistic depth with clarity to ensure cross-disciplinary relevance.

I do have some minor suggestions for consideration to improve fairness along with the quality & accuracy of the review.

(1) The authors use aging studies that compare changes in certain parameters with homeostasis, but to be fair the original study describing the specific phenomenon in homeostasis, should also be cited. For example, the authors discuss the Slc1a3/Dlx1 epidermal stem cell populations and how they change in aging, but it'd be only fair to cite the original papers Sada et al, 2016 (Nat Cell Bio) and Ghuwalewala et al 2024 (Development) that defined and characterized the populations during homeostasis and although not focused on aging they followed their behavior throughout most of the mouse life.

We thank the reviewer for this important point regarding citation fairness and historical context. We agree that when discussing aging-associated changes in defined epidermal progenitor populations, the foundational studies that established these populations during homeostasis should be cited. We have therefore added mentioned papers in the relevant section and we revised the text to more clearly distinguish: (i) population definition/behavior under homeostasis and (ii) subsequent aging-associated shifts in clonal dynamics and regenerative capacity (Page 3, Lines 105-112).

Page 3, Lines 105-112: "In homeostasis, the interfollicular epidermis is sustained by molecularly and spatially distinct basal progenitor pools that can be genetically marked by *Dlx1*^{CreER} and *Slc1a3*^{CreER}, respectively (Sada *et al*, 2016). Notably, the spatial organization of these basal domains in mouse skin shows parallels to human rete ridge and inter-ridge territories. This binary architecture imposes differential environmental exposure across progenitor niches; more deeply embedded progenitors (such as the *Slc1a3*$^{+}$ populations) are proposed to be relatively protected from UV-associated stress compared to their shallower counterparts (Ghuwalewala *et al*, 2024; Ghuwalewala *et al*, 2022; Wang *et al*, 2020)."

This improves precision, fairness, and clarity for readers.

(2) The skin morphology depicted in figures 1&2 is not representative of human skin. Most human skin samples show undulation of the basal layer (rete ridges and inter-ridges) but the top surface is relatively flat. This makes the rete ridge progenitors (Slc1a3+) more deeply embedded in the dermis than the inter-ridge progenitors, and likely their progenitors are more protected from the environment. Ghuwalewala et al (2022, EMBO J) provides evidence that more embedded Slc1a3+ are more protected from UV and given the intense focus of the review on UV exposure, this aspect of skin organization may be interesting to consider along with Slc1a3/Dlx1 populations that were already discussed.

We sincerely thank the reviewer for this insightful comment highlighting both the anatomical accuracy and the conceptual implications of epidermal architecture. In response, we revised Figure 1 and 2 to better reflect human epidermal architecture, incorporating an undulating basal layer (rete ridges/inter-ridges) while maintaining a relatively flat outer surface. This adjustment improves biological realism and aligns the schematics with typical human skin histology.

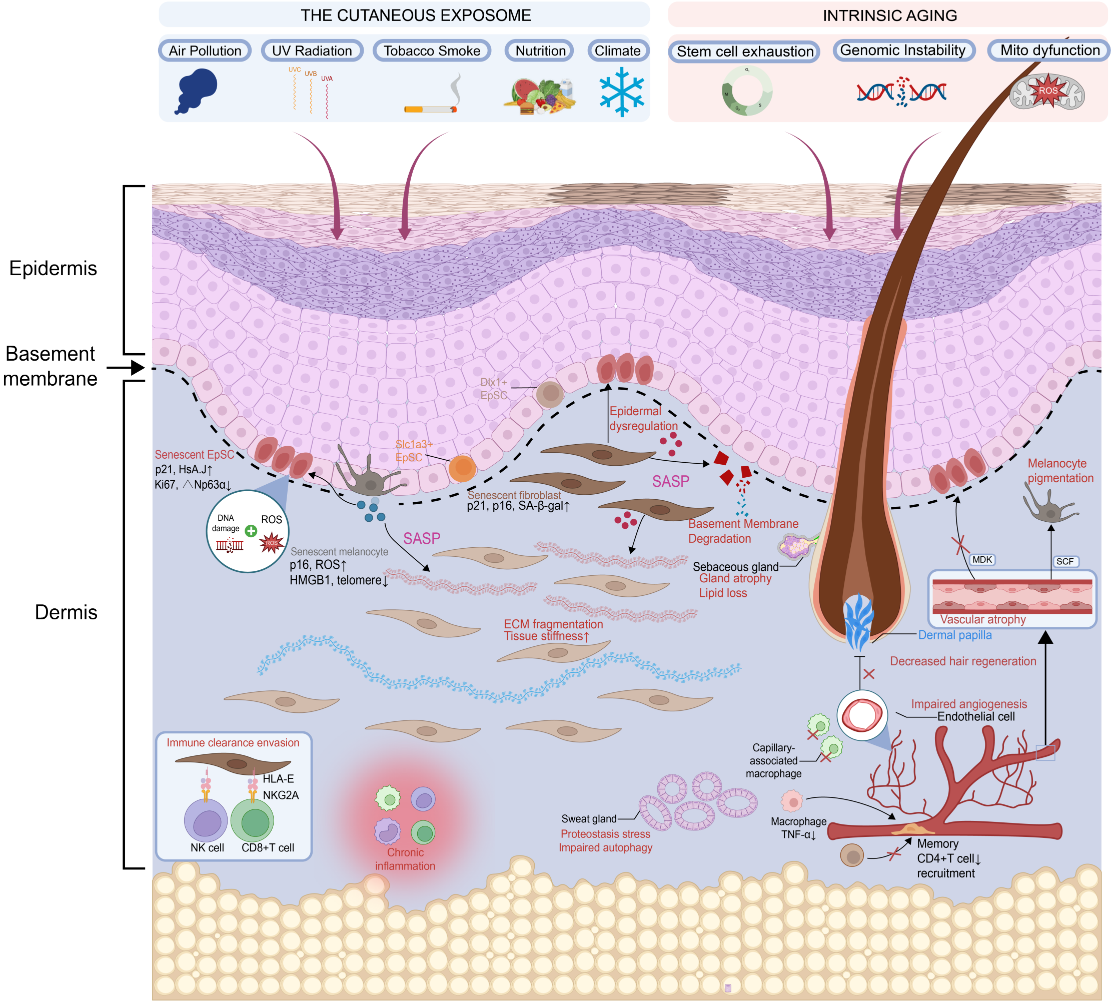

Figure 1. Systemic deterioration of the skin ecosystem during aging. Schematic cross-section of aged human skin illustrating how intrinsic aging mechanisms (stem cell exhaustion, genomic instability, and mitochondrial dysfunction) converge with the cutaneous exposome (air pollution, ultraviolet radiation, tobacco smoke, nutritional factors, and climatic stressors) to disrupt intercellular communication and tissue homeostasis (top). These combined inputs promote multicellular senescence and microenvironmental remodeling—characterized by SASP signaling, basement membrane degradation, ECM fragmentation/stiffening, chronic inflammation, vascular dysfunction, and impaired appendageal regeneration—culminating in tissue-level functional decline across the epidermis, dermis, and skin appendages.

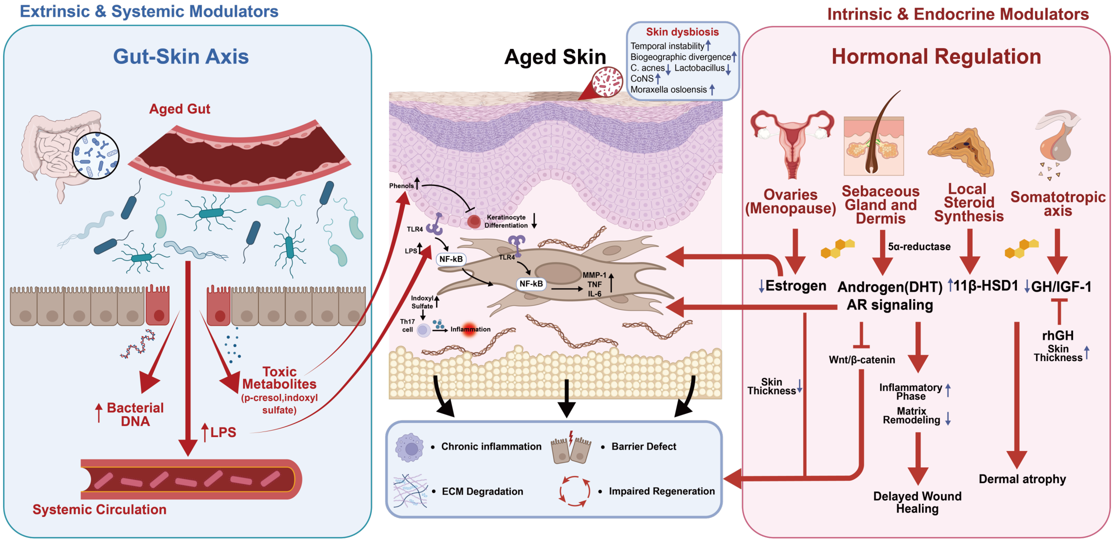

Figure 2. Converging gut–skin microbiome and endocrine mechanisms in skin aging. The figure is arranged as three framed panels to emphasize how extrinsic/systemic and intrinsic/endocrine modulators converge on shared tissue outcomes. Left (Extrinsic & systemic; Gut–Skin Axis): age-associated gut dysbiosis and barrier leakiness promote systemic dissemination of microbial products (e.g., lipopolysaccharide (LPS), bacterial DNA) and metabolites (e.g., p-cresol, indoxyl sulfate), which are strongly shaped by exposome inputs (diet, medications, smoking/pollutants). Middle (Aged skin): circulating cues activate pattern-recognition pathways (e.g., TLR4) in keratinocytes and fibroblasts, driving NF-κB–linked inflammation and matrix metalloproteinase activity (e.g., MMP-1), impairing differentiation and accelerating extracellular matrix (ECM) breakdown; skin dysbiosis may further amplify these responses. Right (Intrinsic & endocrine; Hormonal regulation): reduced estrogen and GH/IGF-1 weaken collagen/skin thickness and regenerative capacity, while increased local androgen signaling (5α-reductase–dependent DHT/androgen receptor (AR)) antagonizes Wnt/β-catenin programs and delays wound healing; elevated 11β-HSD1 enhances glucocorticoid activation and dermal atrophy. Together, these axes bridge intrinsic aging and exposome inputs to drive chronic inflammation, barrier defects, and impaired repair.

In the text, we also incorporated the reviewer's conceptual point that spatial embedding of progenitor niches may modulate exposure burden and vulnerability to environmental stressors, including UV. We cite the mentioned paper in this comment and explicitly note that tissue organization can shape how extrinsic stressors intersect with progenitor programs and aging trajectories (Page 3, Lines 105-112).

Page 3, Lines 105-112: "In homeostasis, the interfollicular epidermis is sustained by molecularly and spatially distinct basal progenitor pools that can be genetically marked by *Dlx1*^{CreER} and *Slc1a3*^{CreER}, respectively (Sada *et al.*, 2016). Notably, the spatial organization of these basal domains in mouse skin shows parallels to human rete ridge and inter-ridge territories. This binary architecture imposes differential environmental exposure across progenitor niches; more deeply embedded progenitors (such as the *Slc1a3*$^{+}$ populations) are proposed to be relatively protected from UV-

associated stress compared to their shallower counterparts (Ghuwalewala *et al.*, 2024; Ghuwalewala *et al.*, 2022; Wang *et al.*, 2020)."

(3) The function of telomeres and telomerase in skin aging have been studied in mice via both deletion of TERC over five mouse generations and overexpression of TERT. Both modifications affect skin stem cells self-renewal and demonstrate contribution to skin aging. Diskeratosis congenita, a telomere length human genetic disease, is mentioned very briefly, but not as a clear illustration of how some aspects of skin aging (hair greying, depigmentation, hair loss) can be linked to telomere length in human.

We sincerely thank the reviewer for this helpful suggestion, which prompted us to clarify both the experimental and clinical evidence linking telomere biology to skin aging. We expanded the section on telomere biology to summarize causal evidence more clearly from mouse models demonstrating effects on skin stem cell self-renewal and degenerative skin/hair phenotypes. We also strengthened the discussion of dyskeratosis congenita as a human telomere biology disorder that provides a clinically relevant illustration linking telomere dysfunction to mucocutaneous abnormalities and hair phenotypes (Page 5, Lines 198-209).

Page 5, Lines 198-209: "Telomere attrition represents a key source of this intrinsic genomic damage, directly linking telomere length to appendage dysfunction. Late-generation *mTR*$^{-/-}$ mice with critically short telomeres develop profound hair/skin degenerative phenotypes (Rudolph *et al*, 1999), whereas epithelial *Tert* gain-of-function promotes progenitor activity and telogen-to-anagen transition (Flores *et al*, 2005; Sarin *et al*, 2005), and conditional telomerase reactivation can partially reverse degenerative changes (Jaskelioff *et al*, 2011; Siegl-Cachedenier *et al*, 2007). In humans, dyskeratosis congenita, a telomere biology disorder, provides a clinical correlate with mucocutaneous abnormalities (including reticulate pigmentation changes) and can include premature hair graying and hair loss (Savage, 2022). Compounding these intrinsic genomic deficits, extrinsic niche deterioration further exacerbates HFSC decline."

These revisions better illustrate how telomere shortening can contribute to skin and hair aging–related features across both experimental models and human disease.

(4) An emerging factor in aging is loss of silencing at transposable elements, which can occur due to increase inflammation or loss of chromatin and youthful epigenetic patterns. While I am not sure how much is known exactly in skin about this, it is a general factor considered in aging. A recent paper from the Ge lab (Cell 2025) showed that loss of a single histone methylase can activate transposons in skin, affecting hair follicle stem cell activation patterns. Although they have not been directly linked this to aging, evidence exists that transposons may be aberrantly activated in inflammatory skin disease, such as psoriasis. The authors consider inflammation a lot in the review, it may be interesting to link it with transposons even if skin currently lacks evidence for the implication of these aging factors.

We thank the reviewer for this forward-looking suggestion. We agree that TE de-repression is an emerging aging mechanism that may intersect with inflammaging and chromatin/epigenetic drift. In the revised manuscript, we added a concise paragraph introducing TE/ERV de-repression in the context of epigenetic drift and inflammaging, while noting that direct evidence in physiological human skin aging remains limited. We incorporated recent mechanistic evidence that loss of a single histone methyltransferase in adult murine skin activates TEs and disrupts HFSC activation with regenerative failure (Lyu et al, 2024), and we also note links between repetitive element/ERV dysregulation and inflammatory skin disease (e.g., psoriasis) (Page 5, Lines 217-234).

Page 5, Lines 217-234: “Finally, emerging work suggests that age-associated epigenetic drift and inflammation may intersect with the loss of silencing at transposable elements (TEs), including endogenous retroviruses (ERVs), thereby activating antiviral pattern-recognition pathways and amplifying type I interferon–linked innate immune signaling via “viral mimicry” (Chiappinelli *et al*, 2015; De Cecco *et al*, 2019; Roulois *et al*, 2015). While direct evidence in human physiological skin aging remains limited, a recent mechanistic study demonstrated that the loss of a single histone methyltransferase (*Setdb1*) in adult murine skin aberrantly activates TEs. This epigenetic disruption alters hair follicle stem cell activation patterns and induces regenerative failure characterized by stem cell exhaustion and hair loss, aspects of which are remarkably reversible with antiviral intervention (Lyu *et al*, 2024). Consistent with the inflammation-centric features of aged skin, repetitive element and ERV dysregulation has also been directly implicated in chronic inflammatory skin diseases such as psoriasis (Krishnan & Kõks, 2023; Molès *et al*, 2005). This supports a plausible interface between chronic inflammation and TE control, providing a novel conceptual framework for understanding epidermal “inflammaging” and stem cell decline.”

Together, this addition provides a cautious conceptual bridge between inflammation, epigenetic instability, and stem cell decline.

(5) Some citations are incomplete - for example it would just say the title of the journal - Nature - but no volume and number and page info. Please double check that all citations are complete and conforming with the journal's format.

We sincerely thank the reviewer for carefully noting this issue and for the helpful suggestion. We have carefully re-checked the full reference list and in-text citations and corrected incomplete entries (including missing volume/issue/page information where applicable), to ensure consistency with the journal’s formatting requirements. These revisions ensure the accuracy of the references and full consistency. Here are the revised references:

Choi E-J, Kil IS, Cho E-G (2020) Extracellular Vesicles Derived from Senescent Fibroblasts Attenuate the Dermal Effect on Keratinocyte Differentiation. *Int J Mol Sci* 21: 1022

Gill D, Parry A, Santos F, Okkenhaug H, Todd CD, Hernando-Herraez I, Stubbs TM,

Milagre I, Reik W (2022) Multi-omic rejuvenation of human cells by maturation phase transient reprogramming. *Elife* 11: e71624
Kim H, Kim J, Lee E, Conklin B, Hou Y, Kim S, Hwang Y, Lee K-B, Kim J (2025a) Alleviation of aging‐related hallmarks in a mouse model of progeria via a nanoparticle‐based artificial transcription factor. *Adv Funct Mater* 35: 2425944
Krishnan VS, Kõks S (2023) Transcriptional Landscape of Repetitive Elements in Psoriatic Skin from Large Cohort Studies: Relevance to Psoriasis Pathophysiology. *Int J Mol Sci* 24: 16725
Kuehne A, Hildebrand J, Soehle J, Wenck H, Terstegen L, Gallinat S, Knott A, Winnefeld M, Zamboni N (2017) An integrative metabolomics and transcriptomics study to identify metabolic alterations in aged skin of humans in vivo. *BMC Genomics* 18: 169
Kuzmich NN, Sivak KV, Chubarev VN, Porozov YB, Savateeva-Lyubimova TN, Peri F (2017) TLR4 Signaling Pathway Modulators as Potential Therapeutics in Inflammation and Sepsis. *Vaccines (Basel)* 5: 34
Manzoni EFM, Pennarossa G, deEguileor M, Tettamanti G, Gandolfi F, Brevini TAL (2016) 5-azacytidine affects TET2 and histone transcription and reshapes morphology of human skin fibroblasts. *Sci Rep* 6: 37017
Mesa KR, O’Connor KA, Ng C, Salvatore SP, Dolynuk A, Lomeli MR, Littman DR (2025) Niche-specific dermal macrophage loss promotes skin capillary ageing. *Nature* 648: 173-181
Mitchell W, Goeminne LJE, Tyshkovskiy A, Zhang S, Chen JY, Paulo JA, Pierce KA, Choy AH, Clish CB, Gygi SP *et al* (2024) Multi-omics characterization of partial chemical reprogramming reveals evidence of cell rejuvenation. *eLife* 12: RP90579
Pei W, Dibeklioglu H, Baltrusaitis T, Tax DMJ (2019) Attended End-to-end Architecture for Age Estimation from Facial Expression Videos. *IEEE Trans Image Process* 29: 1972-1984
Phillip JM, Wu PH, Gilkes DM, Williams W, McGovern S, Daya J, Chen J, Aifuwa I, Lee JSH, Fan R *et al* (2017) Biophysical and biomolecular determination of cellular age in humans. *Nat Biomed Eng* 1: 0093
Thapa RK, Nguyen HT, Jeong J-H, Kim JR, Choi H-G, Yong CS, Kim JO (2017) Progressive slowdown/prevention of cellular senescence by CD9-targeted delivery of rapamycin using lactose-wrapped calcium carbonate nanoparticles. *Sci Rep* 7: 43299
Wang X, Shu X, Huo W, Zou L, Li L (2017c) Efficacy of protein extracts from medium of Adipose-derived stem cells via microneedles on Asian skin. *J Cosmet Laser Ther* 20: 237-244

(6) Figures 1&2 are cited in the intro and that is a nice overview to begin with, but it is not clear where all the things in the figure panels are coming from. It would be nice to cite these figures again in text as the data is discussed in more detail.

We thank the reviewer for this helpful suggestion and for highlighting the importance of clearly linking the overview figures to the detailed discussion in the text. **In the revised manuscript, we added explicit cross-references to Figure 1 and 2 at the**

relevant points throughout the text, so that when specific biological processes or cell types depicted in these schematics are discussed in detail, the corresponding figure citation is provided in the same paragraph. This allows readers to directly map mechanistic discussions (e.g., epidermal stem cell dysfunction, fibroblast senescence and ECM remodeling, vascular and immune microenvironment changes, disrupted crosstalk across epidermis, dermis and appendages, gut–skin axis, and hormonal regulation) back to the visual summaries.

We also refined the **figure legends** (Page 28, Lines 1285-1294; Page 29, Lines 1306-1323) to clarify the conceptual scope of each figure and improve traceability between the panels and the corresponding text. We thank the reviewer again for this constructive suggestion, which helped us improve the clarity and readability of the manuscript.

References

Agius E, Lacy KE, Vukmanovic-Stejic M, Jagger AL, Papageorgiou AP, Hall S, Reed JR, Curnow SJ, Fuentes-Duculan J, Buckley CD *et al* (2009) Decreased TNF-alpha synthesis by macrophages restricts cutaneous immunosurveillance by memory CD4+ T cells during aging. *J Exp Med* 206: 1929-1940

An Y, Wang Q, Gao K, Zhang C, Ouyang Y, Li R, Ma Z, Wu T, Zhou L, Xie Z *et al* (2025) Epigenetic Regulation of Aging and its Rejuvenation. *MedComm* 6: e70369

Bienkowska A, Raddatz G, Söhle J, Kristof B, Völzke H, Gallinat S, Lyko F, Kaderali L, Winnefeld M, Grönniger E *et al* (2023) Development of an epigenetic clock to predict visual age progression of human skin. *Front Aging* 4: 1258183

Bontempi D, Zalay O, Bitterman DS, Birkbak N, Shyr D, Haugg F, Qian JM, Roberts H, Perni S, Prudente V *et al* (2025) FaceAge, a deep learning system to estimate biological age from face photographs to improve prognostication: a model development and validation study. *The Lancet Digital Health* 7: 100870

Bracken OV, De Maeyer RPH, Akbar AN (2025) Enhancing immunity during ageing by targeting interactions within the tissue environment. *Nat Rev Drug Discov* 24: 300-315

Branchet MC, Boisnic S, Frances C, Robert AM (1990) Skin thickness changes in normal aging skin. *Gerontology* 36: 28-35

Chiappinelli KB, Strissel PL, Desrichard A, Li H, Henke C, Akman B, Hein A, Rote NS, Cope LM, Snyder A *et al* (2015) Inhibiting DNA Methylation Causes an Interferon Response in Cancer via dsRNA Including Endogenous Retroviruses. *Cell* 162: 974-986

De Cecco M, Ito T, Petrashen AP, Elias AE, Skvir NJ, Criscione SW, Caligiana A, Brocculi G, Adney EM, Boeke JD *et al* (2019) L1 drives IFN in senescent cells and promotes age-associated inflammation. *Nature* 566: 73-78

Flores I, Cayuela ML, Blasco MA (2005) Effects of telomerase and telomere length on epidermal stem cell behavior. *Science* 309: 1253-1256

Ghadially R, Brown BE, Sequeira-Martin SM, Feingold KR, Elias PM (1995) The aged epidermal permeability barrier. Structural, functional, and lipid biochemical

abnormalities in humans and a senescent murine model. *J Clin Invest* 95: 2281-2290
Ghuwalewala S, Jiang K, Ragi S, Shalloway D, Tumbar T (2024) A transit-amplifying progenitor with biphasic behavior contributes to epidermal renewal. *Development* 151: dev202389
Ghuwalewala S, Lee SA, Jiang K, Baidya J, Chovatiya G, Kaur P, Shalloway D, Tumbar T (2022) Binary organization of epidermal basal domains highlights robustness to environmental exposure. *Embo j* 41: e110488
He X, Gao X, Xie W (2024) Research Progress in Skin Aging and Immunity. *Int J Mol Sci* 25: 4101
Holzscheck N, Falckenhayn C, Söhle J, Kristof B, Siegner R, Werner A, Schössow J, Jürgens C, Völzke H, Wenck H *et al* (2021) Modeling transcriptomic age using knowledge-primed artificial neural networks. *NPJ Aging Mech Dis* 7: 15
James EL, Michalek RD, Pitiyage GN, de Castro AM, Vignola KS, Jones J, Mohney RP, Karoly ED, Prime SS, Parkinson EK (2015) Senescent human fibroblasts show increased glycolysis and redox homeostasis with extracellular metabolomes that overlap with those of irreparable DNA damage, aging, and disease. *J Proteome Res* 14: 1854-1871
James ENL, Bennett MH, Parkinson EK (2018) The induction of the fibroblast extracellular senescence metabolome is a dynamic process. *Sci Rep* 8: 12148
Jankowski CSR, Samarah LZ, MacArthur MR, Mitchell SJ, Weilandt DR, Hunter CJ, Zeng X, McReynolds MR, Rabinowitz JD (2025) Aged mice exhibit widespread metabolic changes but preserved major fluxes. *Cell Metab* 37: 2280-2294.e2284
Jaskelioff M, Muller FL, Paik JH, Thomas E, Jiang S, Adams AC, Sahin E, Kost-Alimova M, Protopopov A, Cadiñanos J *et al* (2011) Telomerase reactivation reverses tissue degeneration in aged telomerase-deficient mice. *Nature* 469: 102-106
Kirkland JL, Tchkonia T (2020) Senolytic drugs: from discovery to translation. *J Intern Med* 288: 518-536
Krishnan VS, Kõks S (2023) Transcriptional Landscape of Repetitive Elements in Psoriatic Skin from Large Cohort Studies: Relevance to Psoriasis Pathophysiology. *Int J Mol Sci* 24: 16725
Laberge RM, Sun Y, Orjalo AV, Patil CK, Freund A, Zhou L, Curran SC, Davalos AR, Wilson-Edell KA, Liu S *et al* (2015) MTOR regulates the pro-tumorigenic senescence-associated secretory phenotype by promoting IL1A translation. *Nat Cell Biol* 17: 1049-1061
Lee YI, Choi S, Roh WS, Lee JH, Kim T-G (2021) Cellular senescence and inflammaging in the skin microenvironment. *Int J Mol Sci* 22: 3849
Lyu Y, Kim SJ, Humphrey ES, Nayak R, Guan Y, Liang Q, Kim KH, Tan Y, Dou J, Sun H *et al* (2024) Stem cell activity-coupled suppression of endogenous retrovirus governs adult tissue regeneration. *Cell* 187: 7414-7432.e7426
Martic I, Papaccio F, Bellei B, Cavinato M (2023) Mitochondrial dynamics and metabolism across skin cells: implications for skin homeostasis and aging. *Front Physiol* 14: 1284410
Molès JP, Tesniere A, Guilhou JJ (2005) A new endogenous retroviral sequence is expressed in skin of patients with psoriasis. *Br J Dermatol* 153: 83-89

Nacarelli T, Lau L, Fukumoto T, Zundell J, Fatkhutdinov N, Wu S, Aird KM, Iwasaki O, Kossenkov AV, Schultz D *et al* (2019) NAD(+) metabolism governs the proinflammatory senescence-associated secretome. *Nat Cell Biol* 21: 397-407
Naharro-Rodriguez J, Bacci S, Hernandez-Bule ML, Perez-Gonzalez A, Fernandez-Guarino M (2025a) Decoding Skin Aging: A Review of Mechanisms, Markers, and Modern Therapies. *Cosmetics* 12: 144
Naharro-Rodriguez J, Bacci S, Hernandez-Bule ML, Perez-Gonzalez A, Fernandez-Guarino M (2025b) Decoding Skin Aging: A Review of Mechanisms, Markers, and Modern Therapies. *Cosmetics* doi: 10.3390/cosmetics12040144 [PREPRINT]
Rittié L, Fisher GJ (2015) Natural and sun-induced aging of human skin. *Cold Spring Harb Perspect Med* 5: a015370
Roig-Rosello E, Rousselle P (2020) The Human Epidermal Basement Membrane: A Shaped and Cell Instructive Platform That Aging Slowly Alters. *Biomolecules* 10: 1607
Roulois D, Loo Yau H, Singhania R, Wang Y, Danesh A, Shen SY, Han H, Liang G, Jones PA, Pugh TJ *et al* (2015) DNA-Demethylating Agents Target Colorectal Cancer Cells by Inducing Viral Mimicry by Endogenous Transcripts. *Cell* 162: 961-973
Rudolph KL, Chang S, Lee HW, Blasco M, Gottlieb GJ, Greider C, DePinho RA (1999) Longevity, stress response, and cancer in aging telomerase-deficient mice. *Cell* 96: 701-712
Sada A, Jacob F, Leung E, Wang S, White BS, Shalloway D, Tumbar T (2016) Defining the cellular lineage hierarchy in the interfollicular epidermis of adult skin. *Nat Cell Biol* 18: 619-631
Sarin KY, Cheung P, Gilison D, Lee E, Tennen RI, Wang E, Artandi MK, Oro AE, Artandi SE (2005) Conditional telomerase induction causes proliferation of hair follicle stem cells. *Nature* 436: 1048-1052
Savage SA (2022) Dyskeratosis congenita and telomere biology disorders. *Hematology Am Soc Hematol Educ Program* 2022: 637-648
Siegl-Cachedenier I, Flores I, Klatt P, Blasco MA (2007) Telomerase reverses epidermal hair follicle stem cell defects and loss of long-term survival associated with critically short telomeres. *J Cell Biol* 179: 277-290
Vukmanovic-Stejic M, Chambers ES, Suárez-Fariñas M, Sandhu D, Fuentes-Duculan J, Patel N, Agius E, Lacy KE, Turner CT, Larbi A *et al* (2018) Enhancement of cutaneous immunity during aging by blocking p38 mitogen-activated protein (MAP) kinase-induced inflammation. *J Allergy Clin Immunol* 142: 844-856
Vukmanovic-Stejic M, Sandhu D, Seidel JA, Patel N, Sobande TO, Agius E, Jackson SE, Fuentes-Duculan J, Suárez-Fariñas M, Mabbott NA *et al* (2015) The Characterization of Varicella Zoster Virus-Specific T Cells in Skin and Blood during Aging. *J Invest Dermatol* 135: 1752-1762
Wang S, Drummond ML, Guerrero-Juarez CF, Tarapore E, MacLean AL, Stabell AR, Wu SC, Gutierrez G, That BT, Benavente CA *et al* (2020) Single cell transcriptomics of human epidermis identifies basal stem cell transition states. *Nat Commun* 11: 4239
Wang TW, Nakanishi M (2025) Immune surveillance of senescence: potential application to age-related diseases. *Trends Cell Biol* 35: 248-257
Wang Y-C, Ma S-H, Chang Y-T, Chen C-C (2025) Clinical evaluation of skin aging: a

systematic review. *Archives of Gerontology and Geriatrics* 139: 105995
Wiley CD, Velarde MC, Lecot P, Liu S, Sarnoski EA, Freund A, Shirakawa K, Lim HW, Davis SS, Ramanathan A *et al* (2016) Mitochondrial Dysfunction Induces Senescence with a Distinct Secretory Phenotype. *Cell Metab* 23: 303-314
Yamazaki S, Nishioka A, Kasuya S, Ohkura N, Hemmi H, Kaisho T, Taguchi O, Sakaguchi S, Morita A (2014) Homeostasis of thymus-derived Foxp3+ regulatory T cells is controlled by ultraviolet B exposure in the skin. *J Immunol* 193: 5488-5497
Zou Z, Long X, Zhao Q, Zheng Y, Song M, Ma S, Jing Y, Wang S, He Y, Esteban CR *et al* (2021) A Single-Cell Transcriptomic Atlas of Human Skin Aging. *Developmental Cell* 56: 383-397.e388

Dear Dr Han,

Thank you for resubmitting the revised review article manuscript, as well as for your patience with our feedback. As indicated earlier, we have asked the experts to reassess your amended manuscript version, and have in the meantime received feedback from both, which I enclose below. As you will see the referees appreciate your amendments to text and figures and are now broadly in favor of publication.

I am thus pleased to let you know that your review article has been provisionally accepted for publication at the EMBO Journal.

We now need you to address a number of formatting points as listed below.

Also, please provide the figures as individual high-resolution image files at your earliest convenience. I will swiftly reach out to our graphics illustrator and his team so they can start preparing the final rendered figure versions of your review.

I look forward to your feedback on this,
and seeing the piece accepted and at production shortly.

Best regards,

Daniel Klimmeck

Daniel Klimmeck, PhD
Senior Editor
The EMBO Journal

Final formatting adjustments required for this review article manuscript:

>> Rename the 'Conflict of Interest' section header to 'DISCLOSURE AND COMPETING INTERESTS STATEMENT'.

>> Please remove figure drafts from the manuscript text upload as individual figure files.
>> Figure legends: remove from the figure files and compile at the end of the manuscript text.
>> Figure callouts: missing for Table 2, please add.

Further information is available in our Guide For Authors: https://www.embopress.org/page/journal/14602075/authorguide

----

Referee #1:

Thank you to the authors for their revision, all comments have been addressed and the manuscript reads well.

Referee #2:

This is a very interesting and useful review and with the new revisions I think it is now ready for publication.

The authors addressed the remaining editorial issues.

2nd Revision - Editorial Decision 14th Apr 2026

Dear Dr Han,

Thank you for submitting the revised version of your review manuscript for consideration by the EMBO Journal.

I have carefully checked your amendments towards the experts' comments and found them to be well addressed and plausibly integrated into the revised text. I am thus very pleased to inform you that your review article has now been accepted for publication in the EMBO Journal.

As mentioned, I have already passed the figures on to our graphics editorial team, who will approach you shortly regarding the versions translated into our journal style.

Your manuscript will be processed for publication by EMBO Press. It will be copy edited and you will receive page proofs prior to publication. Also, you will soon be contacted by Springer Nature to sign your publishing license.

Should you experience any difficulty, please email publishing@embo.org.

If you have any questions, please do not hesitate to contact me or the Editorial Office.

I look forward to progressing swiftly towards online publication of this review article!

with
Best wishes to Peking,

Daniel

Daniel Klimmeck, PhD
Senior Editor
The EMBO Journal
EMBO
Postfach 1022-40
Meyerhofstrasse 1
D-69117 Heidelberg
contact@embojournal.org
Submit at: http://emboj.msubmit.net

*****
Please note that it is The EMBO Journal policy for the transcript of the editorial process (containing referee reports and your response letters) to be published as an online supplement to each paper. If you should prefer removal of any referee-only figures included in the point-by-point response(s), e.g. because they may still be used for future publication or because they have been reproduced from published work by others, please do let us know immediately via response email.
More information is available here: https://link.springer.com/partners/embo-press/editorial-policies#Peer%20review
